# Stepwise Diagnostic Product Ions Filtering Strategy for Rapid Discovery of Diterpenoids in *Scutellaria barbata* Based on UHPLC-Q-Exactive-Orbitrap-MS

**DOI:** 10.3390/molecules27238185

**Published:** 2022-11-24

**Authors:** Xinhua Zhou, Xu Chen, Liping Fan, Huirong Dong, Yan Ren, Xiangming Chen

**Affiliations:** School of Pharmacy, Binzhou Medical University, Yantai 264003, China

**Keywords:** diterpenoids, UHPLC-Q-Exactive-Orbitrap-MS, stepwise diagnostic product ions, *Scutellaria barbata*

## Abstract

Diterpenoids are considered the major bioactive components in *Scutellaria barbata* to treat cancer and inflammation, but few comprehensive profiling studies of diterpenoids have been reported. Herein, a stepwise diagnostic product ions (DPIs) filtering strategy for efficient and targeted profiling of diterpenoids in *Scutellaria barbata* was developed using UHPLC-Q-Exactive-Orbitrap-MS. After UHPLC-HRMS/MS analysis of six diterpenoid reference standards, fragmentation behaviors of these references were studied to provide DPIs. Then, stepwise DPIs filtering aimed to reduce the potential interferences of matrix ions and achieve more chromatographic peaks was conducted to rapidly screen the diterpenoids. The results demonstrated that stepwise DPIs were capable of simplifying the workload in data post-processing and the effective acquisition of low abundance compounds. Subsequently, DPIs and MS/MS fragment patterns were adopted to identify the targeted diterpenoids. As a result, 381 diterpenoids were unambiguously or tentatively identified, while 141 of them with completely new molecular weights were potential new diterpenoids for *Scutellaria barbata*. These results demonstrate that the developed stepwise DPIs filtering method could be employed as an efficient, reliable, and valuable strategy to screen and identify the diterpenoid profile in *Scutellaria barbata*. This might accelerate and simplify target constituent profiling from traditional Chinese medicine (TCM) extracts.

## 1. Introduction

*Scutellaria barbata* (Ban-Zhi-Lian in Chinese), the dried whole plant of *Scutellaria barbata* D. Don, has been documented in the Chinese Pharmacopoeia since 1985. It has been popularly used for heat clearing, detoxifying, diuresis promoting, cancer treatment, and inflammation elimination with anti-tumor and anti-inflammatory pharmacological activities [1,2,3]. The most remarkable pharmacological activity of *Scutellaria barbata* is its anti-tumor activity [4,5,6,7,8]. Although the complicated chemical composition of *Scutellaria barbata* mainly includes diterpenoids, flavonoids, alkaloids, polysaccharides, volatile oils, and steroids [1,9,10,11], the components with antitumor activity in *Scutellaria barbata* are diterpenoids [12,13] and flavonoids [9,14]. A new diterpenoid named barbatin H, together with fifteen known analogues isolated from *Scutellaria barbata,* showed anti-tumor activity against four human tumor cell lines including LoVo, MCF-7, SMMC-7721, and HCT-116 cells by the MTT method in vitro [14]. Studies aimed to systematically discover and characterize flavonoids in *Scutellaria barbata* using LC-HRMS have been widely reported [15,16]. In the preliminary research, we also developed a strategy integrating parent ions list-modified mass defect filtering-diagnostic product ions for rapid screening and systematic characterization of flavonoids in *Scutellaria barbata* [16]. However, there have been a few studies on the systematic characterization of diterpenoids in *Scutellaria barbata* up to now. Therefore, it is necessary to develop a new method for the comprehensive analysis of diterpenes in *Scutellaria barbata*.

Tandem high-resolution mass spectrometry, especially tandem Q-Exactive orbitrap mass spectrometry (Q-Exactive Orbitrap/MS) with remarkable sensitivity coupled with UHPLC, is widely used in the chemical composition analysis of traditional Chinese medicine (TCM) [17,18,19,20]. With the advantage of sensitively acquiring more data, the subsequent disadvantage of this technology is that a large amount of MS data needs a tediously long time for data analysis. In order to achieve the purpose of saving time and improving speed, great efforts have been made to develop more effective MS data post-processing methods such as mass defect filtering (MDF) [21,22,23,24], neutral loss filtering (NLF) [25,26], extraction ion chromatography (EIC) [16,27,28], and so on. These data post-processing techniques simplify the characterization process [21,22,23,24,25,26,27,28], but the exclusion of false positive results and structure confirmation still depends on diagnostic product ions (DPIs) produced in the MS fragmentation.

In order to exclude more false positive results, a method named key ion filtering (KIF), which relies on the main DPIs for filtering, has been developed [29]. The KIF method can be applied to exclude the majority of irrelevant ions automatically and simplify the data. The single chromatographic peak filtered by this method actually used the parent ion peak as the background, and the convex part of the peak was a superposition of multiple secondary fragment ions. However, this method only paid attention to the fragment ion peak with high abundance and ignored the fragment ion peak with low abundance, especially when the fragment ion peak intensity/parent ion peak intensity was less than 3. Therefore, in order to ensure the effective discovery of low abundance fragment ion peaks, we proposed a stepwise DPIs filtering mode with multiple time windows that could effectively magnify the time, distinguishing more low abundance fragment ion peaks from the background (parent ion peak).

Herein, a new strategy based on stepwise DPIs filtering was proposed and applied to rapidly discover diterpenoids in *Scutellaria barbata* using UHPLC-Q-Exactive Orbitrap/MS. After the MS and MS^2^ data of the *Scutellaria barbata* extract and reference substances were acquired via UHPLC-Q-Exactive Orbitrap/MS, the DPIs were analyzed according to the MS fragmentation pathway and the structural characteristics of diterpenoids. Then, DPIs were used for both MS data filtering and structure confirmation to tentatively identify a total of 381 diterpenoids. This strategy can be used to rapidly discover diterpenoids from *Scutellaria barbata* and be extended to detecting other compounds in TCM.

## 2. Results and Discussion

### 2.1. Optimization of MS Conditions

**Figure 1:**
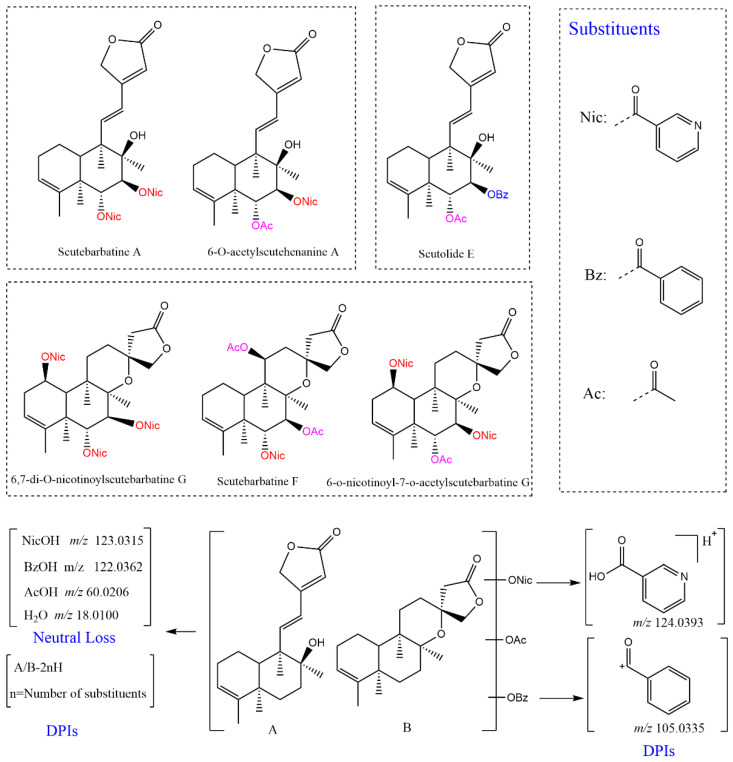
Chemical structures and fragment information of six reference substances under the positive ion mode.

It is difficult to obtain MS^2^ data of diterpenoids due to their low concentration and the possibility of being covered up by the co-outflowed flavonoids or other compounds with high content. To cover more compounds, the 15 most abundant predictors were selected to acquire MS/MS spectrum for each scan cycle. Six reference substances (see Figure 1) were used to compare MS responsivity and the amount of fragment information under the positive and negative ion modes. The results showed that the above indices were better in the positive ion mode. For the optimization of higher energy collision induced dissociation (HCD) energy, the comparison of normalized collision energy (NCE) between 35 eV (the stepped NCE was 15, 35, 55 eV) and 50 eV (the stepped NCE was 25, 50, 75 eV) showed that no significant difference was obtained. The NCE at 50 eV was selected randomly. Under the above settings, we comprehensively collected the MS/MS spectra of diterpenoids in *Scutellaria barbata*.

### 2.2. DPIs Investigation of Diterpenoids

#### 2.2.1. Structure Analysis and Subtype Attribution

**Figure 2:**
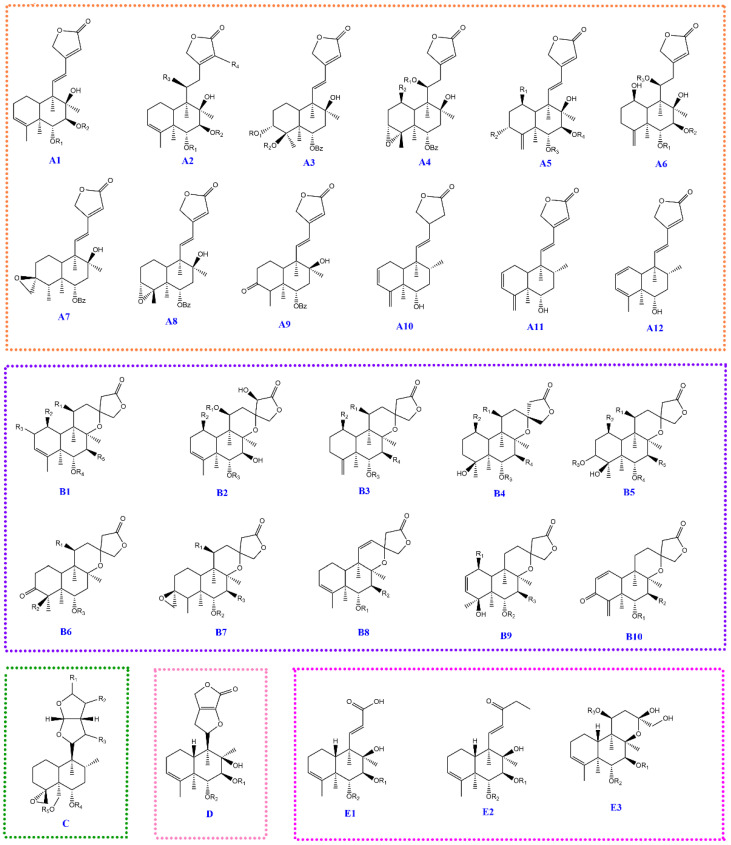
Twenty-seven subtypes of diterpenoids in *Scutellaria barbata* whose substituents mainly included the nicotinoyloxy, benzoyloxy, acetyl, hydroxyl C_4_H_7_O_2_, C_5_H_7_O_3_, C_7_H_11_O_4_, and C_9_H_15_O_4_ groups.

By investigating the diterpenoids discovered thus far in *Scutellaria barbata*, the diterpenoids showed diversity and complexity, although most of them contained 20 carbon atoms. These can be divided into five classes [1,30,31,32]: (A) furan-2(5H)-one type diterpenoid; (B) 2,6-dioxaspiro [4.5]decan-3-one type diterpenoid; (C) hexahydrofuro[2,3-b]furan type diterpenoid; (D) 3,6-dihydrofuro[3,4-b]furan-4(2H)-one type diterpenoid; (E) degraded diterpenoid). According to the mother nucleus structure, twenty-seven subtypes were formed, as demonstrated in Figure 2. The substituents added in the diterpenoid backbone mainly included nicotinoyloxy (ONic), benzoyloxy (OBz), acetyl (OAc), and hydroxyl groups. In addition, C_4_H_7_O_2_, C_5_H_7_O_3_, C_7_H_11_O_4_, and C_9_H_15_O_4_ were unusual substituents.

#### 2.2.2. DPIs Investigation

We were lucky to obtain six reference substances gifted by Shengjun Dai. Among them, scutebarbatine A, scutolide E, and 6-O-acetylscutehenanine A belonged to category A1, while 6,7-di-O-nicotinoylscutebarbatine G, scutebarbatine F, and 6-O-nicotinoyl-7-O-acetylscutebarbatine G belonged to category B1. They were mixed together for analysis by UHPLC-HRMS/MS with a final concentration of 5 μg/mL for each compound. The fragmentation pathway and DPIs information for six reference standards are shown in Figure 1 (more details in Figures S1–S6 and Table S1). For compounds in categories A1 and B1, they mainly cleaved the nicotinoyloxy and benzoyloxy groups to form protonated nicotinic acid (C_6_H_6_O_2_N, 124.0393 Da) and benzoyl ion (C_7_H_5_O, 105.0335 Da) in the MS^2^ spectrum under the positive ion mode. Due to other subtype diterpenoids having the same substituents as the above two subtypes, they would produce the same key fragment ions at *m*/*z* 124.0393 and *m*/*z* 105.0335 as above. Hence, *m*/*z* 124.0393 and *m*/*z* 105.0335 were used as DPIs for filtering diterpenoids of all types.

Parent nuclear ions also formed after the substitutions of nicotinoyloxy, benzoyloxy, acetyl, and hydroxyl groups were eliminated. For example, a neutral loss of 123.0315 [C_6_H_5_O_2_N, nicotinic acid (NicA)], 122.0326 [C_7_H_6_O_2_, benzoic acid (BzA)], 18.0100 (H_2_O), and 60.0206 [C_2_H_4_O_2_, acetic acid (AcA)] can be observed to generate the parent nuclear ion at *m*/*z* 313.1798 (C_20_H_25_O_3_) in the MS^2^ spectrum of the above reference substances. In addition, the diterpenoids also contained other substituents such as C_4_H_7_O_2_, C_5_H_7_O_3_, C_7_H_11_O_4_, and C_9_H_15_O_4_, according to the literature reports [31]. Hence, these compounds could lose the above substituents and generate a neutral loss of 88.0519 Da (C_4_H_8_O_2_), 116.0468 Da (C_5_H_8_O_3_), 160.0730 Da (C_7_H_12_O_4_), and 188.1041 Da (C_9_H_15_O_4_), respectively, which are conducive to rapid structural confirmation. With the difference in the number of nicotinoyloxy, benzoyloxy, acetyl, hydroxyl, and other groups, the parent nuclear ions formed would be different. By changing the number of substituents, the parent nuclear ions database of other subtypes of diterpenoids were predicted, as shown in Table 1. After DPIs filtering (*m*/*z* 124.0393 and 105.0335) of diterpenoids, the above parent nuclear ions were directly used for structure analysis.

### 2.3. Research Strategy for the Characterization of Diterpenoids

**Figure 3:**
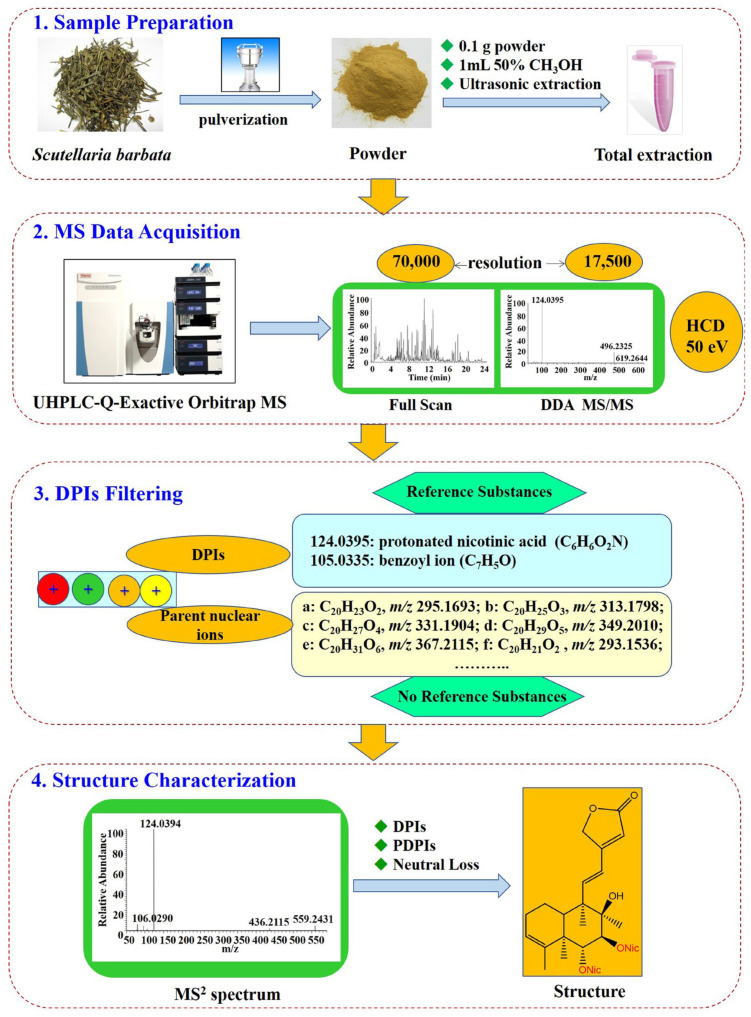
A flowchart for the stepwise DPIs filtering strategy developed for systematic exposure and characterization of diterpenoids in *Scutellaria barbata*.

**Figure 4:**
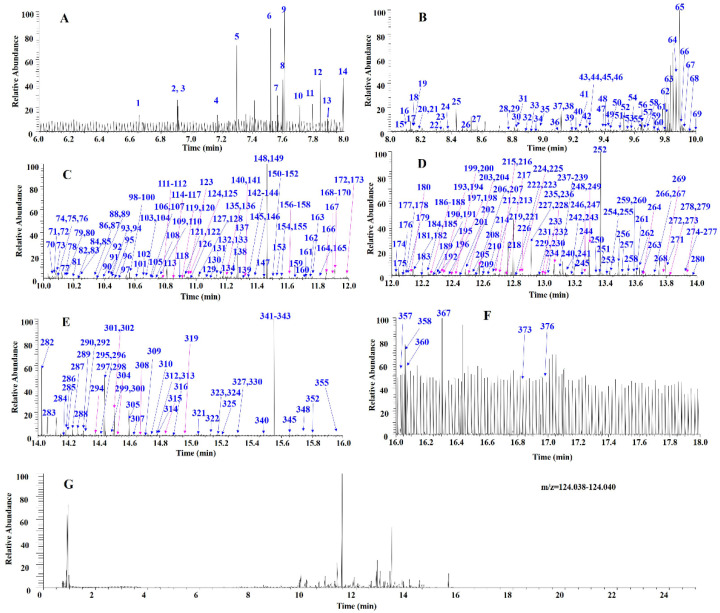
The stepwise DPIs filtering chromatograms of *m*/*z* 124.0393 (**G**) with six time-windows set as follows: (**A**) 6.0–8.0 min; (**B**) 8.0–10.0 min; (**C**) 10.0–12.0 min; (**D**) 12.0–14.0 min; (**E**) 14.0–16.0 min; (**F**) 16.0–18.0 min.

**Figure 5:**
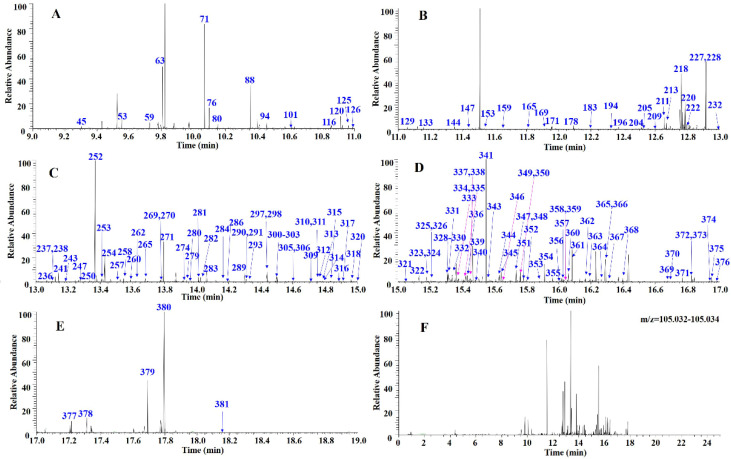
The stepwise DPIs filtering chromatograms of *m*/*z* 105.0335 (**F**) with five time-windows set as follows: (**A**) 9.0–11.0 min; (**B**) 11.0–13.0 min; (**C**) 13.0–15.0 min; (**D**) 15.0–17.0 min; (**E**) 17.0–19.0 min.

The following stepwise DPIs filtering strategy was used for the characterization of diterpenoids in *Scutellaria barbata* (Figure 3). First, the mass spectra fingerprint of *Scutellaria barbata* and six references were obtained by UHPLC-Q-Exactive Orbitrap/MS. Second, we acquired their HRMS/MS spectra and analyzed their MS fragment information to summarize the DPIs based on the references. Third, stepwise DPIs filtering was used to filter the diterpenoids. The retention time was split into two minute time-windows to enlarge the chromatogram in each 2 min. The target fragment ion peaks were obtained after subtracting the background (here, it was the total ion peak). The stepwise DPIs filtering chromatograms of *m*/*z* 124.0393 with six time-windows was set as follows: (A) 6.0–8.0 min; (B) 8.0–10.0 min; (C) 10.0–12.0 min; (D) 12.0–14.0 min; (E) 14.0–16.0 min; (F) 16.0–18.0 min (Figure 4), while the stepwise DPIs filtering chromatograms of *m*/*z* 105.0335 with five time-windows was set as follows: (A) 9.0–11.0 min; (B) 11.0–13.0 min; (C) 13.0–15.0 min, (D) 15.0–17.0 min; (E) 17.0–19.0 min (Figure 5). By using the stepwise DPIs ion extraction (Xcalibur software) of *m*/*z* 124.0393 and *m*/*z* 105.0335 Da, the fragment ion peaks were recognized. Finally, the target compounds were identified and confirmed by analyzing their MS and MS/MS spectra.

In the stepwise DPIs method, the retention time range from 6 to 19 min was divided equally into several two-minute time-windows to generate DPIs chromatograms. The background ions had the same retention time interval and similarity abundance. Then, the ions with increased abundance and those not coincident with the background ions were judged as the target ions. The results showed that the stepwise DPIs method with two-minute time-windows may more fully expose the diterpene peaks, especially the low abundance compounds and even search for the target ion peaks hidden in the background compared with the DPIs method alone. Taking 124.0393 Da filtering as an example, 10, 46, and 175 target ions were found when the relative intensity was higher than 10%, 5%, and 1%, respectively. The DPIs method could not focus on the ions with an abundance less than 1%, and the ions screened were far lower than the 327 target ions found in the six time-window stepwise DPIs filtering chromatogram.

### 2.4. Systematic Characterization of Diterpenoids in Scutellaria Barbata

After the above not blindly filtering the MS/MS spectra of all the determined ions, we quickly captured the target diterpenoids. As a result, a total of 381 diterpenoids were picked out and recognized. Their structural elucidations proceeded according to their structural types and the MS/MS fragmentation pathways. The characterized diterpenoids are shown in Table S2. It is worth noting that different subtype diterpenoids had the same base peak in the MS^2^ spectrum due to the same substituent. Moreover, diterpenoids of several subtypes produced the same parent nuclear ions after fragmentation. For example, fourteen subtypes of diterpenoids including A1, A2, A3, A4, A5, A8, A9, B1, B3, B4, B5, B6, B8, and C can all produce parent nuclear ions C_20_H_25_O_3_ at *m*/*z* 313.1798. Therefore, DPIs can only be used to determine whether the compounds were diterpenes and what substituents were connected to the diterpenes, but the specific category of diterpenes and the connection position of the substituents could not be determined. Among the 381 diterpenes found above, 141 compounds had completely new molecular weights compared with the literature. Structural elucidation of some representative cases could be seen in the following examples.

Compounds 128, 138, 148, 160, and 175 showed fragment ions at *m*/*z* 124.0393 (C_6_H_6_O_2_N) and 313.1798 (C_20_H_25_O_3_), which were undoubtedly designated as 6-O-nicotinoyl-7-O-acetylscutebarbatine G, scutebarbatine F, scutebarbatine A, 6,7-di-O-nicotinoylscutebarbatine G, 6-O-acetylscutehenanine A, and scutolide E by comparing the retention time, accurate molecular weight, and DPIs of the reference substance. Compound 293 could be identified as scutolide E through a comparison with the standard, which showed molecular weight at *m*/*z* 105.0335 (C_7_H_5_O) and 313.1798 (C_20_H_25_O_3_).

Compounds 227, 260, 271, 285, 291, 303, and 311 showed an [M+H]^+^ ion at *m*/*z* 618.2698 (C_35_H_40_O_9_N). In the MS^2^ spectra, their protonated molecular ions generated a series of product ions at *m*/*z* 124.04 (C_6_H_6_O_2_N^+^), 105.03 (C_7_H_5_O^+^), 295.17 ([M+H-AcA-BzA-NicA-H_2_O]^+^), 313.18 ([M+H-AcA-BzA-NicA]^+^), 436.21 ([M+H-AcA-BzA]^+^), and 558.25 ([M+H-AcA]^+^), formed by the loss of NicA, BzA, AcA, and H_2_O. In comparison with the MS fragmentation patterns obtained from the reference standards, compounds 227, 260, 271, 285, 291, 303, and 311 were reasonably characterized as C_20_H_25_O_3_-NicA-BzA-AcA.

Compound 288 produced an [M+H]^+^ ion at *m*/*z* 738.3273 (C_43_H_48_O_10_N). In the MS^2^ spectra, the most intense ion appeared at *m*/*z* 147.0441 (C_9_H_7_O_2_^+^), which indicated the existence of a new substituent. The ions at *m*/*z* 124.0394 (C_6_H_6_O_2_N^+^), 105.0338 (C_7_H_5_O^+^), 293.1534 ([M+H-C_9_H_6_O_2_-BzA-3H_2_O-NicA]^+^), 311.1683 ([M+H-C_9_H_6_O_2_-BzA-2H_2_O-NicA]^+^), 329.1743 ([M+H-C_9_H_6_O_2_-BzA-H_2_O-NicA]^+^), and 452.2061 ([M+H-C_9_H_6_O_2_-BzA-H_2_O]^+^) could also be detected, which were formed by the loss of NicA, BzA, C_9_H_6_O_2_, and H_2_O. In them, a new neutral loss of 146.0362 Da (C_9_H_6_O_2_) was observed. According to the above data, compound 288 was tentatively identified as C_20_H_27_O_5_-NicA-BzA-C_9_H_6_O_2_.

Compounds 249, 259, 264, and 277 generated an [M+H]^+^ ion at *m*/*z* 612.2803 (C_33_H_42_O_10_N). They generated a series of fragment ions at *m*/*z* 124.04 (C_6_H_6_O_2_N^+^), 295.17 ([M+H-AcA-BzA-NicA-H_2_O]^+^), 313.18 ([M+H-AcA-BzA-NicA]^+^), 295.17 ([M+H-C_5_H_8_O_3_-2H_2_O-NicA]^+^), 313.18 ([M+H-C_5_H_8_O_3_-H_2_O-NicA]^+^), 331.19 ([M+H-C_5_H_8_O_3_-NicA]^+^), 436.21 ([M+H-C_5_H_8_O_3_-H_2_O]^+^), 454.22 ([M+H-C_5_H_8_O_3_]^+^), and 552.26 ([M+H-AcA]^+^), which were formed by the neutral loss of Nic, C_5_H_8_O_3_, and H_2_O. The substituent of 115.0390 (C_5_H_7_O_3_) has been reported in *Scutellaria barbata* [31]. In comparison with the MS fragmentation data, compounds 249, 259, 264, and 277 were tentatively identified as C_20_H_27_O_4_-NicA-C_5_H_7_O_3_.

## 3. Materials and Methods

### 3.1. Reagents and Materials

The aboveground part of *Scutellaria barbata* was collected from Henan Province, China and was identified by Doctor Shaoping Wang. The six standards of 6,7-di-O-nicotinoylscutebarbatine G, scutebarbatine F, 6-O-nicotinoyl-7-O-acetylscutebarbatine G, scutebarbatine A, scutolide E, and 6-O-acetylscutehenanine A were kindly gifted by Professor Shengjun Dai, School of Pharmacy, Yantai University. The structures of all of the standards are presented in Figure 1. Methanol and acetonitrile of LC-MS grade were purchased from ACS (Houston, TX, USA). Ultrapure deionized water was produced by using a Milli-Q water purification system (Millipore, Billerica, MA, USA). Ethanol and formic acid (analytical grade) were obtained from Sinopharm Chemical Reagent Co. Ltd. (Shanghai, China).

### 3.2. Preparation Standard and Sample Solutions

Six reference substances of 1 mg were prepared by dissolving in 10 mL methanol to make individual stock solutions. Then, each stock solution was mixed to make a final mixed solution with the concentration of 5 μg/mL for each compound.

The dried crude materials were pulverized into powder and passed through a 40 mesh screen. The powder form was accurately weighed and dissolved in a 50% (*v*/*v*) methanol and water solvent system by ultrasonicating for 60 min at 100 Hz. The solution obtained was centrifuged through a 0.45 μm membrane filter and stored at 4 °C before being injected into the UHPLC/HRMS system for analysis.

### 3.3. UHPLC-Q-Exactive Orbitrap/HRMS

A Q-Exactive Orbitrap/HRMS (Thermo Fisher Scientific Inc., Grand Island, NY, USA) system was coupled with an Ultimate 3000 system (Dionex, Sunnyvale, CA, USA) via an electrospray ionization interface. Samples were carried out on a Waters HSS T3 (100 × 2.1 mm, 1.8 μm). The column oven temperature was maintained at 30 °C. The mobile phase consisting of methanol (A) and 0.1% formic acid (B) was delivered at a flow rate of 0.35 mL·min^−1^. A gradient program was used as follows: 2% A at 0–1 min, 2–25% A at 1–4 min, 25–95% A at 4–20 min, 95% A at 20–22 min, 95%–2% A at 22–23 min, and 2% A at 23–25 min. The injection volume was 2 μL.

The ESI source was operated in the positive ion mode, and the Q-Exactive Orbitrap mass operation parameters were set as follows: spray voltage, 3500 V; sheath gas (N_2_) flow rate, 35 Arb; aux gas (N_2_) flow rate, 15 Arb; capillary temperature, 350 °C; source heater temperature, 300 °C. Full-scan analyses were conducted and the spectra were recorded in the range *m*/*z* 100–1000 with a resolution of 70,000. Fragmentation modes of HCD were employed in data-dependent acquisition and fifteen ions with the highest abundance detected in each MS scan were selected for the determination of their MS^2^ fragmentation with a resolution of 17,500. NCE was set at 50 eV with the stepped normalized collision energy of 25, 50, and 75 eV.

Data recording and processing were uniformly processed by Xcalibur 3.0 software (Thermo Fisher Scientific). Elemental composition prediction was set as the following settings: elements in use, C 0–50, H 0–80, N 0–10, O 0–20; mass tolerance <5 ppm; ring double-bond equivalent (RDB eq), 1–30.

### 3.4. Stepwise DPIs Filtering

The diterpenoids in *Scutellaria barbata* can be divided into five categories A–E and further classified into twenty-seven subtypes due to the diversity of the mother nucleus structure (see Figure 2). The acquisition of DPIs relies on the MS data of a large number of references covering each category or even the subtype of components. The difficulty in obtaining the reference substances, the diversity of skeletons, and substituents for diterpenoids in *Scutellaria barbata* make it difficult to obtain the DPIs of each category compound. Fortunately, most diterpenoids have similar substituents and these substituents form the DPIs of diterpenoids. Hence, the DPIs of partial categories without a reference substance can be inferred from the obtained DPIs of other categories.

Stepwise DPIs filtering divides the retention time into two-minute time-windows. After being magnified on the software for 2 min, the parent ion peak (background) was regularly arranged at equal intervals (about 0.02 min apart). Therefore, the parent ion can be clearly distinguished from the filtered DPIs fragment ion peak, and the extremely low abundance fragment ion peak hidden in the background can still be well recognized.

## 4. Conclusions

In this work, a stepwise DPIs filtering strategy was successfully developed for the rapid discovery of diterpenoids in *Scutellaria barbata* based on HPLC-Q-Exactive-Orbitrap-MS. After the MS and MS^2^ data of the reference substances acquired, the DPIs were analyzed according to the characteristic fragmentation pathways of the reference standards. Then, stepwise DPIs filtering was conducted to obtain the diterpenoids. Compared with the KIF method, the stepwise DPIs filtering with multiple time windows could enlarge the spectrum, search for a greater number of potential diterpenoids from the background even if they were hidden in the background, and increase the coverage of constituent filtering. As a result, a total of 381 diterpenoids were characterized or tentatively identified including 141 compounds with a new molecular mass never reported in *Scutellaria barbata*. In this study, the diterpenoids in *Scutellaria barbata* have been systematically reported for the first time. The stepwise DPIs strategy might accelerate and simplify the diterpenoid profiling in *Scutellaria barbata* and could provide a routine approach for the analysis of diterpenoids in other herbal medicines or preparations.

## Figures and Tables

**Table 1:** The DPIs for twenty-seven subtypes of diterpenoids in *Scutellaria barbata*.

Subtypes	Type of Substituents	DPIs
A1	1. R_1_, R_2_ *≠* H	a, b
	2. R_1_ = H or R_2_ = H	a, b, c
	3. R_1_, R_2_ = H	a, b, c, d
A2	1. R_1_, R_2_, R_3_ *≠* H, R_4_ = H	a, b
	2. R_1_, R_2_, R_3_ *≠* H, R_4_ = OH	f, g, h
	3. R_1_, R_3_ *≠* H, R_2_, R_4_ = H or R_2_, R_3_ *≠* H, R_1_, R_4_ = H or R_1_, R_2_ *≠* H, R_3_ = H, R_4_ = OH	a, b, c
	4. R_1_, R_3_ *≠* H, R_2_ = H, R_4_ = OH or R_2_, R_2_ *≠* H, R_1_ = H, R_4_ = OH	f, g, h, i
	5. R_1_, R_2_ *≠* H, R_3_ = H, R_4_ = H	j, k
	6. R_1_ *≠* H, R_2_, R_3_, R_4_ = H or R_2_ *≠* H, R_1_, R_3_, R_4_ = H	j, k, l
	7. R_1_ *≠* H, R_2_, R_3_ = H, R_4_ = OH; or R_2_ *≠* H, R_1_, R_3_ = H, R_4_ = OH	a, b, c, d
	8. R_1_, R_2_, R_3_, R_4_ = H	j, k, l, m
	9. R_1_, R_2_, R_3_, = H, R_4_ = OH	a, b, c, d, e
A3	1. R_1_ = H, R_2_ *≠* H or R_2_ = H, R_1_ *≠* H	a, b, c
	2. R_1_, R_2_ = H	a, b, c, d
A4	1. R_1_ *≠* H, R_2_ = H	b, c
	2. R_1_ *≠* H, R_2_ = OH	g, h, i
A5	1. R_1_ = H, R_2_ = OH, R_3_, R_4_ *≠* H or R_2_ = H, R_1_ = OH, R_3_, R_4_ *≠* H	f, g, h
	2. R_1_, R_2_ = H, R_3_, R_4_ *≠* H	a, b
A6	1. R_1_, R_2_, R_3_ *≠* H	g, h
A7	-	o, p
A8, A9	-	b, c
A10, A12	-	u, v
A11	-	j, k
B1	1. R_1_, R_3_ = H, R_4_ *≠* H, R_2_, R_5_ = OR, R *≠* H or R_2_, R_3_ = H, R_4_ *≠* H, R_1_, R_5_ = OR, R *≠* H	a, b
	2. R_1_, R_3_ = H, R_4_ *≠* H, R_5_ = OH, R_2_ = OR, R *≠* H or R_2_, R_3_ = H, R_4_ *≠* H, R_5_ = OH, R_1_ = OR, R *≠* H	a, b, c
	3. R_1_ = OR, R *≠* H, R_2_, R_3_, R_4_ = H, R_5_ = OH	a, b, c, d
	4. R_1_, R_2_, R_3_ = H, R_4_ *≠* H, R_5_ = OR, R *≠* H or R_1_, R_3_, R_5_ = H, R_4_ *≠* H, R_2_ = OR, R *≠* H	j, k
	5. R_2_, R_5_ = OH, R_3_ = H, R_4_ *≠* H, R_1_ = OR, R *≠* H	f, g, h, i
	6. R_2_ = OH, R_3_ = H, R_4_ *≠* H, R_1_, R_5_ = OR, R *≠* H or R_1_ = H, R_3_ = OH R_4_ *≠* H, R_2_, R_5_ = OR, R *≠* H	f, g, h
B2	1. R_1_, R_3_ *≠* H, R_2_ = H	f, g, h, i
	2. R_1_, R_3_ *≠* H, R_2_ = OH	n, o, p, q
B3	1. R_1_ = OR, R *≠* H, R_2_ = H, R_3_ *≠* H, R_4_ = OH or R_2_ = OR, R *≠* H, R_1_ = H, R_3_ *≠* H, R_4_ = OH or R_4_ = OR, R *≠* H, R_2_ = H, R_3_ *≠* H, R_1_ = OH	a, b, c
	2. R_1_, R_2_, R_4_ = OR, R *≠* H, R_3_ *≠* H	f, g
B4	1. R_1_ = H, R_2_, R_4_ = OR, R *≠* H, R_3_ *≠* H	a, b, c
B5	1. R_1_, R_3_, R_5_ = H, R_2_ = OR, R *≠* H, R_4_ *≠* H or R_2_, R_3_, R_5_ = H, R_1_ = OR, R *≠* H, R_4_ *≠* H	a, b, c, d
	2. R_2_, R_5_ = H, R_1_ = OR, R *≠* H, R_3_, R_4_ *≠* H	a, b, c
B6	1. R_1_ = OR, R *≠* H, R_2_ = H, R_3_ *≠* H	b, c
	2. R_1_ = OR, R *≠* H, R_2_ = OH, R_3_ *≠* H	g, h, i
B7	1. R_1_ = OR, R *≠* H, R_2_ *≠* H, R_3_ *=* H	o, p
	2. R_1_ = OR, R *≠* H, R_2_ *≠* H, R_3_ = OH	r, s, t
B8	1. R_1_ *≠* H, R_2_ = OR, R *≠* H	a, b
B9	1. R_1_, R_3_ = OR, R *≠* H, R_2_ *≠* H	f, g, h
B10	1. R_1_ *≠* H, R_2_ = OR, R *≠* H	n, o
C	1. R_1_ = OR, R *≠* H, R_2_, R_3_ = H, R_4_, R_5_ *≠* H	k
	2. R_1_, R_2_, R_3_ = H, R_4_, R_5_ *≠* H	v
	3. R_2_ = OR, R *≠* H, R_1_ = H, R_3_ = OH, R_4_, R_5_ *≠* H	b, c
D	1. R_1_, R_2_, R_3_ *≠* H	w, x
E1	1. R_1_, R_2_ *≠* H	y, z, a’
E2	1. R_1_, R_2_ *≠* H	b’, c’
E3	1. R_1_ = H, R_2_ *≠* H	g, h, i
	2. R_1_, R_2_ *≠* H	h, i

a: C_20_H_23_O_2_, *m*/*z* 295.1693; b: C_20_H_25_O_3_, *m*/*z* 313.1798; c: C_20_H_27_O_4_, *m*/*z* 331.1904; d: C_20_H_29_O_5_, *m*/*z* 349.2010; e: C_20_H_31_O_6_, *m*/*z* 367.2115; f: C_20_H_21_O_2_, *m*/*z* 293.1536; g: C_20_H_23_O_3_, *m*/*z* 311.1642; h: C_20_H_25_O_4_, *m*/*z* 329.1747; i: C_20_H_27_O_5,_ *m*/*z* 347.1853; j: C_20_H_25_O_2_, *m*/*z* 297.1849; k: C_20_H_27_O_3_, *m*/*z* 315.1955; l: C_20_H_29_O_4_, *m*/*z* 333.2060; m: C_20_H_31_O_5_, *m*/*z* 351.2166; n: C_21_H_25_O_2_, *m*/*z* 309.1849; o: C_21_H_27_O_3_, *m*/*z* 327.1955; p: C_21_H_29_O_4_, *m*/*z* 345.2060; q: C_21_H_31_O_5_, *m*/*z* 363.2166; r: C_21_H_25_O_3_, *m*/*z* 325.1798; s: C_21_H_27_O_4_, *m*/*z* 343.1904; t: C_21_H_29_O_5_, *m*/*z* 361.2010; u: C_20_H_27_O_2_, 299.2006; v: C_20_H_29_O_3_, 317.2111; w: C_18_H_23_O_2_, *m*/*z* 271.1693; x: C_18_H_25_O_3_, *m*/*z* 289.1798; y: C_17_H_19_O, *m*/*z* 239.1430; z: C_17_H_21_O_2_, *m*/*z* 257.1536; a’: C_17_H_23_O_3_, *m*/*z* 275.1642; b’: C_19_H_25_O, *m*/*z* 269.1900; c’: C_19_H_27_O_2_, *m*/*z* 287.2006.

## Data Availability

Not applicable.

## Supplementary Materials

The following supporting information can be downloaded at: https://www.mdpi.com/article/10.3390/molecules27238185/s1, Figure S1: Production formation pathways for 6-O-nicotinoyl-7-O-acetylscutebarbatine G, Figure S2: Product ion formation pathways for scutebarbatine F, Figure S3: Product ion formation pathways for 6,7-di-O-nicotinoylscutebarbatine G, Figure S4: Product ion formation pathways for scutebarbatine A, Figure S5: Product ion formation pathways for 6-O-acetylscutehenanine A, Figure S6: Product ion formation pathways for scutolide E, Table S1: UHPLC-HRMS/MS analysis of six reference standards, Table S2: Systematic exposure and characterization of 381 diterpenoids in *Scutellaria barbata* using the stepwise DPIs filtering strategy.

## Sample Availability

Samples of the compounds are not available from the authors.
