# Supplementary material for: Stepwise Diagnostic Product Ions Filtering Strategy for Rapid Discovery of Diterpenoids in Scutellaria barbata Based on UHPLC-Q-Exactive-Orbitrap-MS
Source: Molecules. 2022 Nov 24;27(23):8185. doi: 10.3390/molecules27238185 (PMC9736491; doi:10.3390/molecules27238185)
Supplement: Supplementary file 1 [file molecules-27-08185-s001.zip › molecules-2049555-supplementary.pdf]

Supplementary material

Article

Stepwise Diagnostic Product Ions Filtering Strategy for Rapid Discovery of Diterpenoids in *Scutellaria barbata* Based on UHPLC-Q-Exactive-Orbitrap-MS

Xinhua Zhou †, Xu Chen †, Liping Fan, Huirong Dong, Yan Ren * and Xiangming Chen

School of Pharmacy, Binzhou Medical University, Yantai 264003, China
* Correspondence: renyan198251@163.com
† These authors contributed equally to this work.

Abbreviations Used

DPIs, diagnostic product ions; UHPLC-HRMS/MS, ultra high-performance liquid chromatography/tandem high-resolution mass spectrometry; Q-Exactive Orbitrap/MS, tandem Q-Exactive orbitrap mass spectrometry; TCM, traditional Chinese medicine; MDF, mass defect filtering; NLF, neutral loss filtering; EIC, extraction ion chromatography; KIF, key ion filtering; HCD, higher energy collision induced dissociation; NCE, normalized collision energy; TIC, total ion chromatogram.

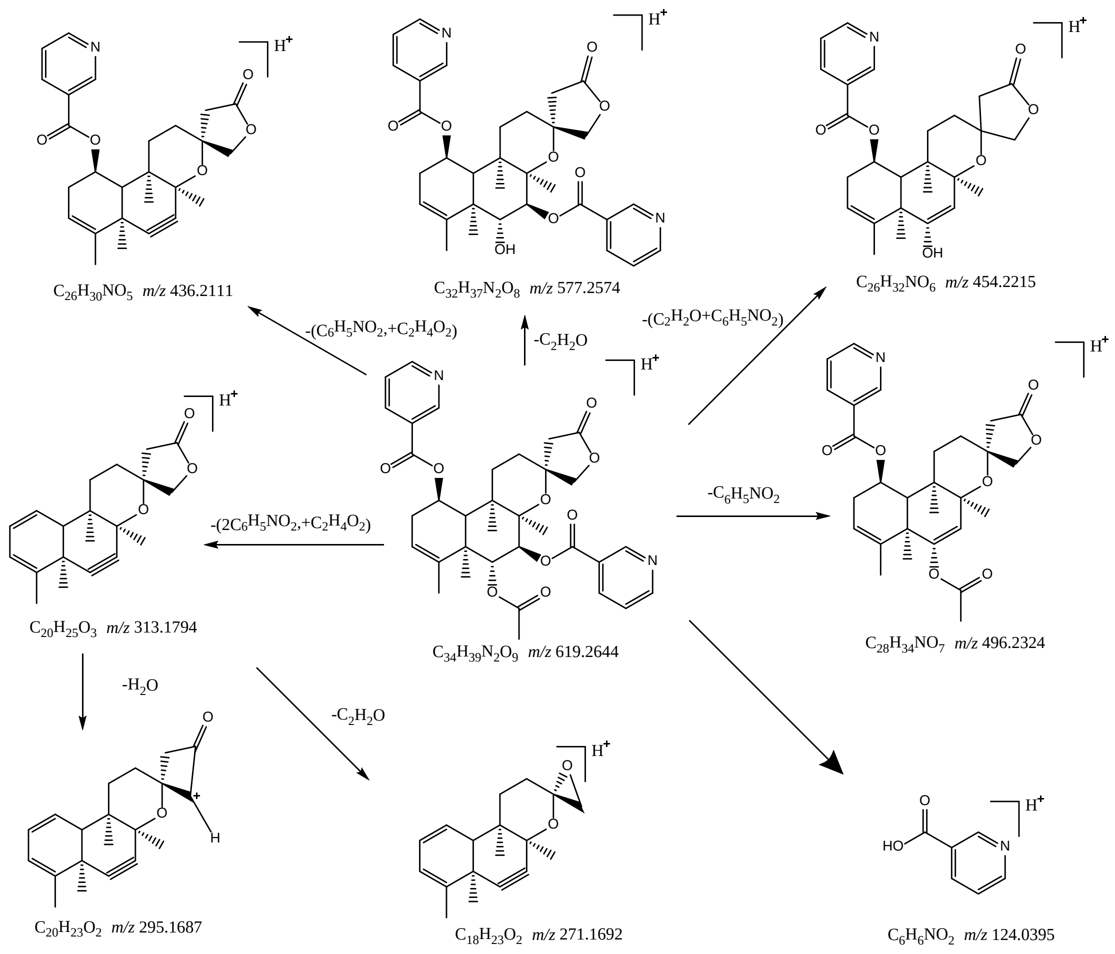

Figure S1 Product ion formation pathways for 6-O-nicotinoyl-7-O-acetylscutebarbatine G.

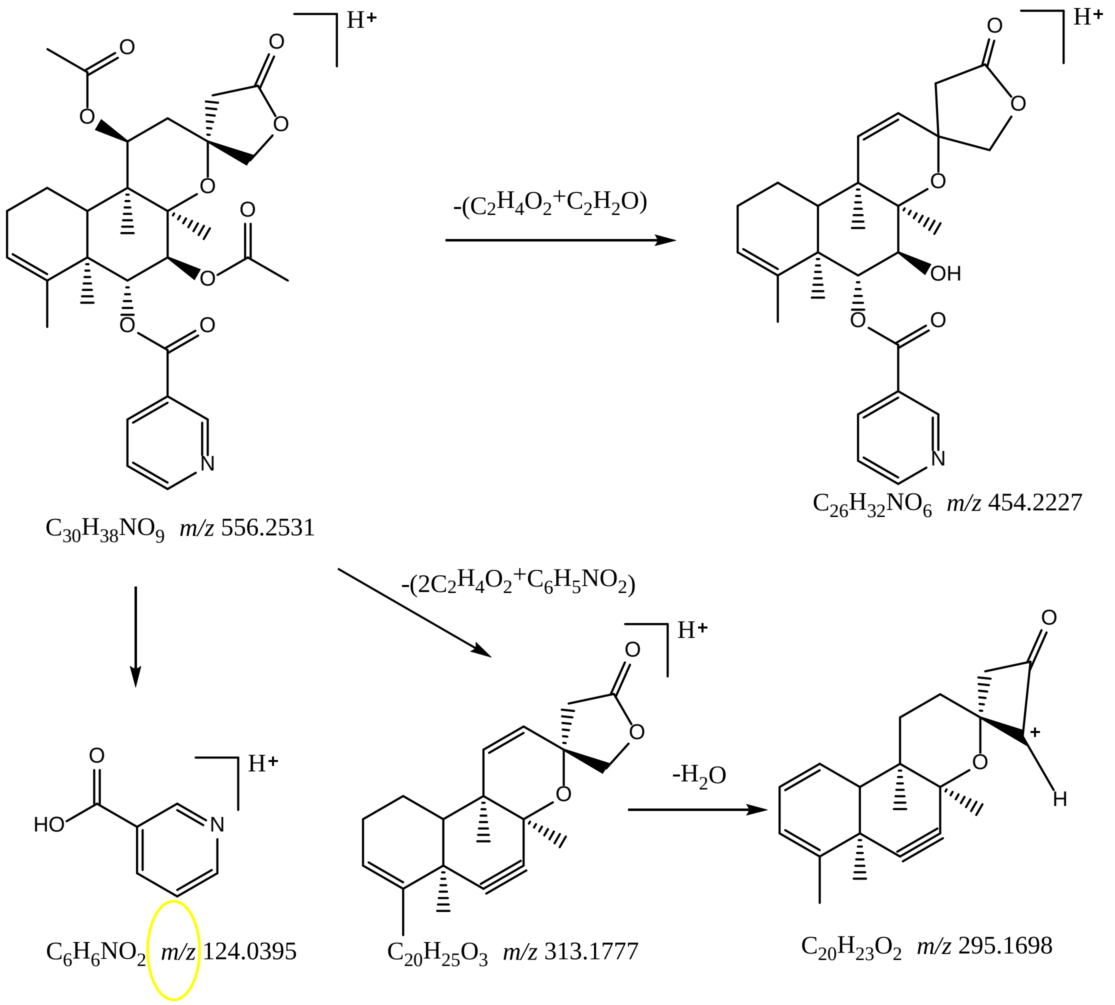

Figure S2 Product ion formation pathways for scutebarbatine F.

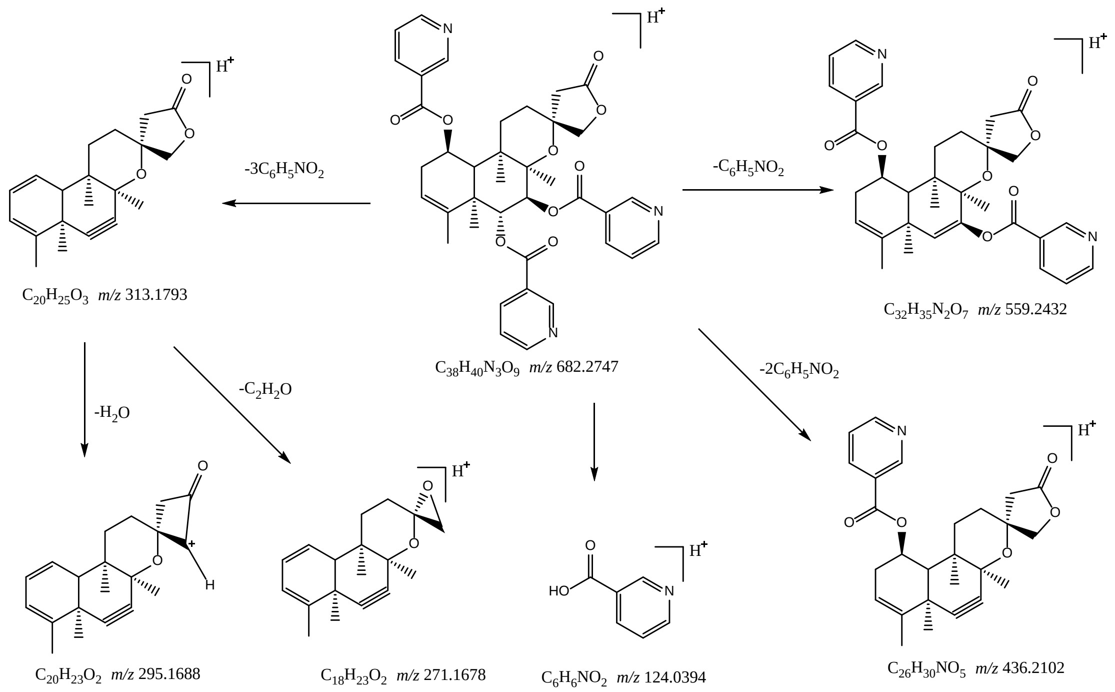

Figure S3 Product ion formation pathways for 6,7-di-O-nicotinoylscutebarbatine G.

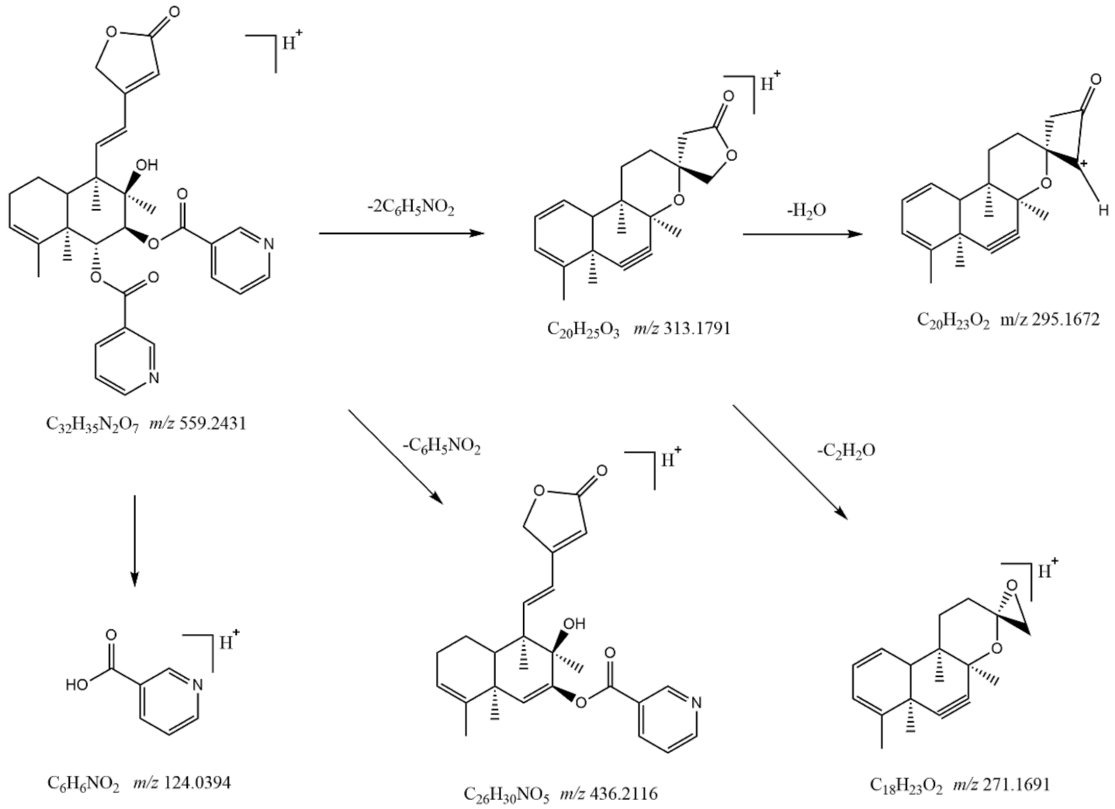

Figure S4 Product ion formation pathways for scutebarbatine A.

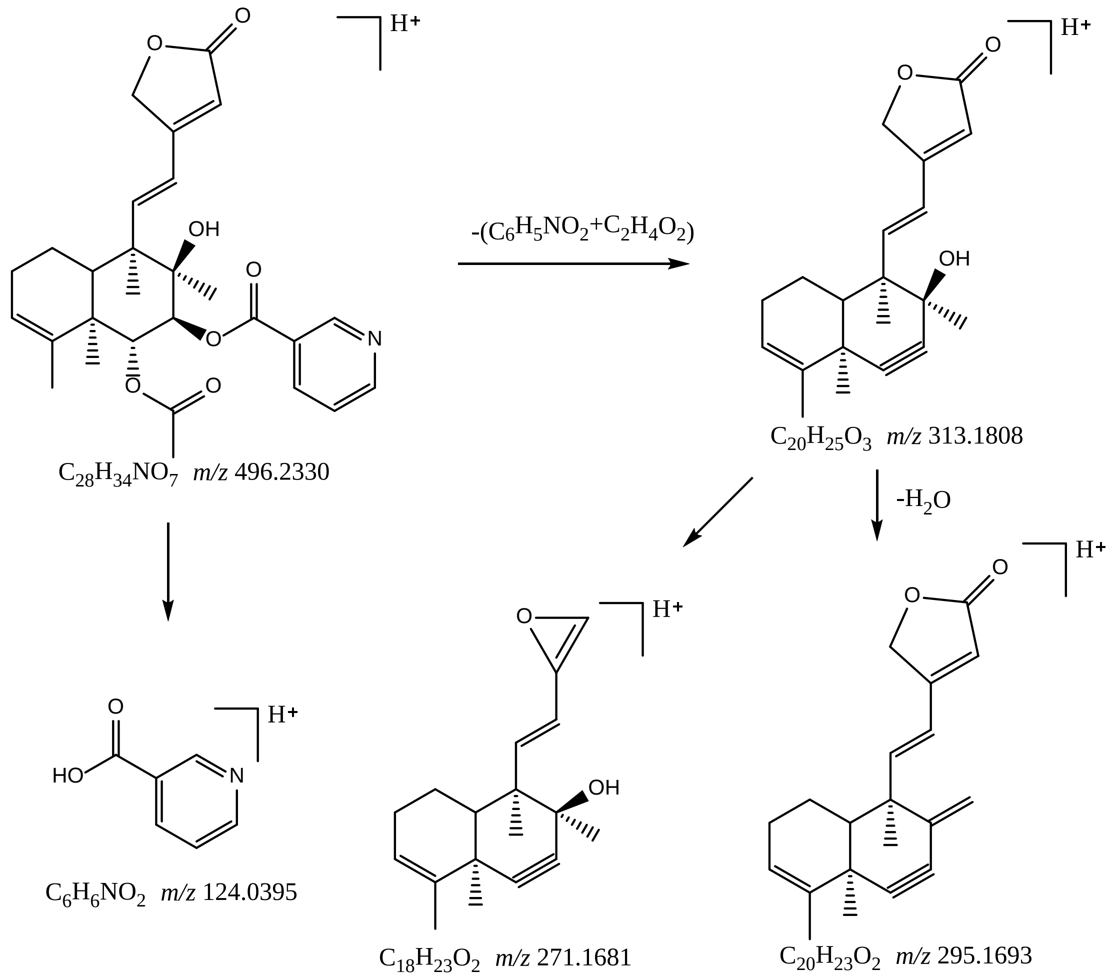

Figure S5 Product ion formation pathways for 6-O-acetylscutehenanine A.

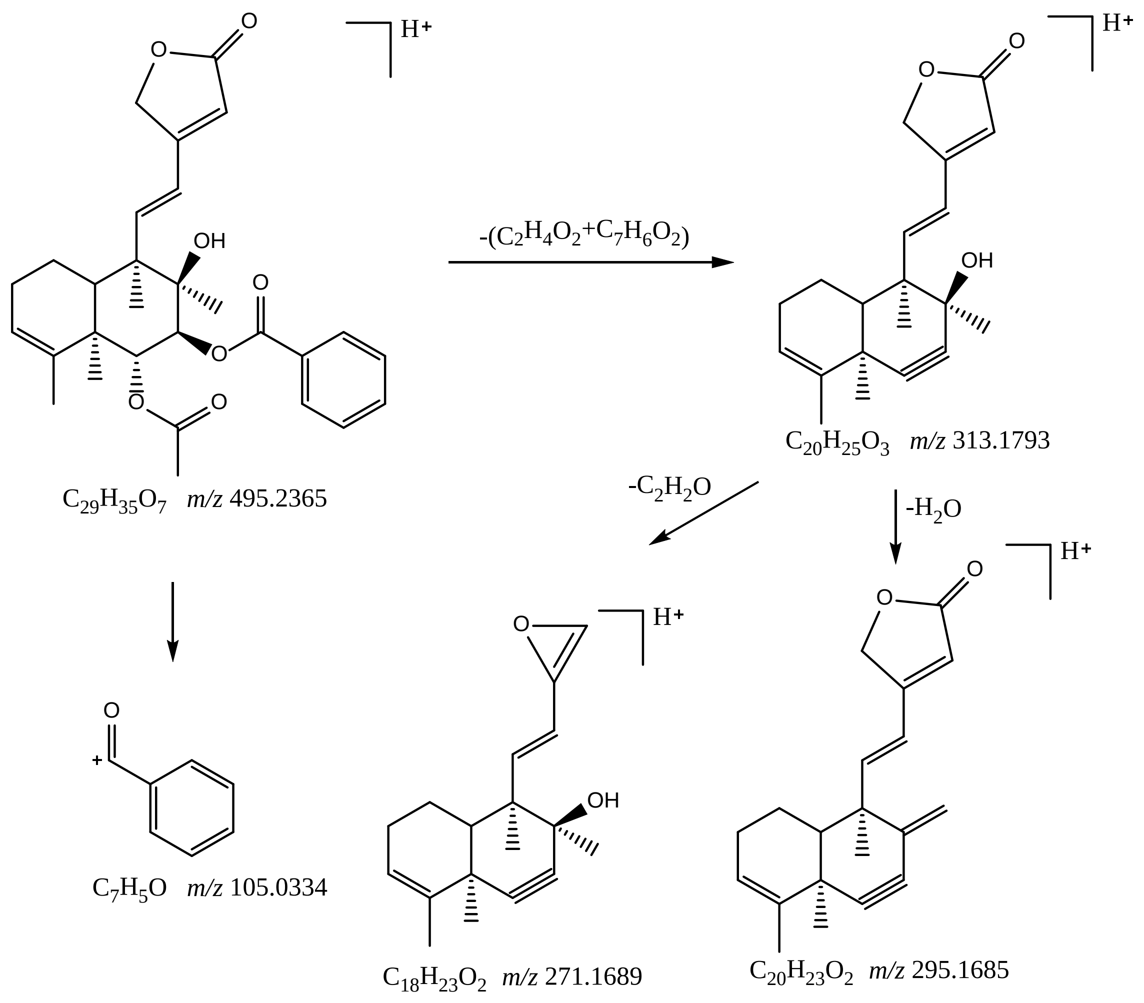

Figure S6 Product ion formation pathways for scutolide E.

Table S1. UHPLC-HRMS/MS analysis of six reference standards.

NO.	T_R (min)	Compounds	Experimental Mass *m/z*	Theoretical Mass *m/z*	Molecular formula	Error (ppm)	MS^2 Fragmentation
1	11.06	6-O-nicotinoyl-7-O-acetylscutebarbatine G	619.2644	619.2650	$C_{34}H_{39}O_9N_2$	-0.980	124.0395,496.2324,313.1794,577.2524,454.2215,295.1687,436.2111, 271.1692
2	11.24	Scutebarbatine F	556.2531	556.2541	$C_{30}H_{38}O_9N$	-1.812	124.0395,313.1777,295.1698,454.2227
3	11.47	Scutebarbatine A	559.2431	559.2439	$C_{32}H_{35}O_7N_2$	-1.391	124.0394,436.2116,295.1672,313.1791,271.1691
4	11.67	6,7-di-O-nicotinoylscutebarbatine G	682.2747	682.2759	$C_{38}H_{40}O_9N_3$	-1.768	124.0394,313.1793,436.2102,295.1688,559.2432,271.1678
5	12.07	6-O-acetylscutehenanine A	496.2324	496.2330	$C_{28}H_{34}O_7N$	-2.292	124.0395,496.2324,295.1693,313.1808,271.1681
6	14.38	Scutolide E	495.2365	495.2377	$C_{29}H_{35}O_7$	-2.483	105.0334,313.1793,295.1685,271.1689

Table S2. Systematic exposure and characterization of 381 diterpenoids in *Scutellaria barbata* using stepwise DPIs filtering strategy.

NO.	T_R (min)	Experimental Mass m/z	Theoretical Mass m/z	Precursors/ Adduct	Molecular formula	Error (ppm)	MS[2] Fragmentation	Identification
1[a]	6.66	546.2332	546.2334	$[M+H]^+$	$C_{28}H_{36}O_{10}N$	-0.307	124.0395,468.2010,486.2155,345.1702,327.1579,309.1479	$C_{20}H_{27}O_6$-NicA-AcA
2	6.91	544.2172	544.2177	$[M+H]^+$	$C_{28}H_{34}O_{10}N$	-0.951	124.0394,484.1961,466.1859,343.1543,325.1432	$C_{20}H_{25}O_6$-NicA-AcA
3[a]	6.91	488.2270	488.2279	$[M+H]^+$	$C_{26}H_{34}O_8N$	-1.830	124.0394,311.1629,329.1737	$C_{20}H_{29}O_6$-NicA
4[a]	7.17	678.3101	678.3120	$[M+H]^+$	$C_{34}H_{48}O_{13}N$	-2.826	124.0395,297.1847,315.1952,438.2271,498.2482,660.3021	$C_{20}H_{27}O_3$-NicA-AcA-Glc
5[a]	7.30	546.2321	546.2334	$[M+H]^+$	$C_{28}H_{36}O_{10}N$	-2.330	124.0394,327.1579,345.1688,454.2193	$C_{20}H_{27}O_6$-NicA-AcA
6	7.52	472.2323	472.2330	$[M+H]^+$	$C_{26}H_{34}O_7N$	-1.437	124.0395,295.1683,313.1800,331.1911,454.2201,472.2323	$C_{20}H_{29}O_5$-NicA
7	7.56	575.2382	575.2388	$[M+H]^+$	$C_{32}H_{35}O_8N_2$	-1.030	124.0394,293.1526,311.1628,329.1733,434.1948,452.2060	$C_{20}H_{25}O_4$-2NicA
8	7.60	530.2385	530.2385	$[M+H]^+$	$C_{28}H_{36}O_9N$	0.079	124.0395,293.1537,311.1631,329.1733,470.2187	$C_{20}H_{27}O_5$-NicA-AcA
9	7.61	472.2324	472.2330	$[M+H]^+$	$C_{26}H_{34}O_7N$	-1.226	124.0395,313.1795,331.1891,472.2324	$C_{20}H_{29}O_5$-NicA
10[a]	7.74	512.2269	512.2279	$[M+H]^+$	$C_{28}H_{34}O_8N$	-1.939	124.0395,293.1525,311.1640,329.1733,434.1927,452.2059	$C_{20}H_{25}O_4$-NicA-AcA
11[a]	7.79	546.2335	546.2334	$[M+H]^+$	$C_{28}H_{36}O_{10}N$	0.233	124.0395,327.1600,345.1697,486.2130,546.2335	$C_{20}H_{27}O_6$-NicA-AcA
12	7.84	472.2323	472.2330	$[M+H]^+$	$C_{26}H_{34}O_7N$	-1.437	124.0394,295.1686,313.1798,331.1896	$C_{20}H_{29}O_5$-NicA
13	7.89	530.2404	530.2385	$[M+H]^+$	$C_{28}H_{36}O_9N$	3.662	124.0394,293.1533,311.1634,329.1748,434.1935,452.2018	$C_{20}H_{27}O_5$-NicA-AcA
14	7.99	530.2386	530.2385	$[M+H]^+$	$C_{28}H_{36}O_9N$	0.362	124.0395,293.1528,311.1638,329.1752,434.1942,452.2067,470.2174 ,530.2386	$C_{20}H_{27}O_5$-NicA-AcA
15[a]	8.07	590.2943	590.2960	$[M+H]^+$	$C_{31}H_{44}O_{10}N$	-2.834	124.0395,297.1842,315.1952,438.2270,456.2387,498.2475	$C_{20}H_{27}O_3$-NicA-AcA-X1
16	8.10	530.2379	530.2385	$[M+H]^+$	$C_{28}H_{36}O_9N$	-1.053	124.0395,293.1523,311.1619,329.1743,347.1851,434.1947,452.2065 ,470.2164	$C_{20}H_{27}O_5$-NicA-AcA
17	8.12	593.2477	593.2494	$[M+H]^+$	$C_{32}H_{37}O_9N_2$	-2.793	124.0395,311.1648,329.1743,452.2070,470.2170	$C_{20}H_{27}O_5$-2NicA
18	8.13	573.2225	573.2231	$[M+H]^+$	$C_{32}H_{33}O_8N_2$	-1.121	124.0395,309.1495,327.1581,450.1905	$C_{20}H_{23}O_4$-2NicA
19[a]	8.14	692.2893	692.2913	$[M+H]^+$	$C_{34}H_{46}O_{14}N$	-2.862	124.0395,311.1638,329.1745,347.1836,452.2060,470.2156,512.2277 ,530.2385	$C_{20}H_{25}O_4$-NicA-AcA-Glc
20[a]	8.17	560.2477	560.2490	$[M+H]^+$	$C_{29}H_{38}O_{10}N$	-2.361	124.0395,291.1375,309.1481,327.1589,345.1685,468.2025,500.2265	$C_{20}H_{25}O_5$-NicA-X1

							,528.2182	
21[a]	8.17	528.2211	528.2228	$[M+H]^+$	$C_{28}H_{34}O_9N$	-3.234	124.0395,309.1475,327.1582,345.1692,450.1920,468.2002	$C_{20}H_{25}O_5$-NicA-AcA
22[a]	8.30	418.2576	418.2588	$[M+H]^+$	$C_{24}H_{36}O_5N$	-2.868	124.0395,259.2049,277.2145,400.2478	$C_{18}H_{31}O_3$-NicA
23	8.33	514.2433	514.2435	$[M+H]^+$	$C_{28}H_{36}O_8N$	-0.473	124.0395,106.0291,313.1794,331.1899,436.2108,454.2225	$C_{20}H_{27}O_4$-NicA-AcA
24[a]	8.38	488.2274	488.2279	$[M+H]^+$	$C_{26}H_{34}O_8N$	-1.011	124.0395,329.1743,347.1848	$C_{20}H_{29}O_6$-NicA
25[a]	8.43	516.2585	516.2592	$[M+H]^+$	$C_{28}H_{38}O_8N$	-1.343	124.0394,297.1841,315.1953,333.2069,420.2147,438.2270,456.2365	$C_{20}H_{29}O_4$-NicA-AcA
26	8.50	472.2328	472.2330	$[M+H]^+$	$C_{26}H_{34}O_7N$	-0.379	124.03944,313.17731,454.22015	$C_{20}H_{29}O_5$-NicA
27	8.57	575.2381	575.23879	$[M+H]^+$	$C_{32}H_{35}O_8N_2$	-1.204	124.0395,293.1532,311.1638,416.1852,434.1958,452.2059,	$C_{20}H_{25}O_4$-2NicA
							539.2149,557.2290	
28[a]	8.78	651.2531	651.2549	$[M+H]^+$	$C_{34}H_{39}O_{11}N_2$	-2.666	124.0394,291.1370,309.1484,327.1584,432.1794,450.1895,492.2016	$C_{20}H_{25}O_5$-2NicA-AcA
							,510.2094,555.2121,573.2220,633.2426	
29	8.79	572.2479	572.2490	$[M+H]^+$	$C_{30}H_{38}O_{10}N$	-1.962	124.0394,293.1523,311.1635,329.1731,512.2258	$C_{20}H_{25}O_4$-NicA-2AcA
30[a]	8.82	512.2275	512.2279	$[M+H]^+$	$C_{28}H_{34}O_8N$	-0.768	124.0394,293.1518,311.1638,329.1751,452.2065	$C_{20}H_{25}O_4$-NicA-AcA
31	8.83	472.2325	472.2330	$[M+H]^+$	$C_{26}H_{34}O_7N$	-1.014	124.0394,331.1911,472.2323	$C_{20}H_{29}O_5$-NicA
32	8.88	577.2538	577.2544	$[M+H]^+$	$C_{32}H_{37}O_8N_2$	-1.113	124.0395,295.1691,313.1794,436.2113,454.2216	$C_{20}H_{27}O_4$-2NicA
33	8.92	574.2632	574.2647	$[M+H]^+$	$C_{30}H_{40}O_{10}N$	-2.565	124.0395,295.1685,313.1799,436.2117,454.2242	$C_{20}H_{27}O_4$-NicA-2AcA
34	8.95	472.2324	472.2330	$[M+H]^+$	$C_{26}H_{34}O_7N$	-1.226	124.0395,313.1794,331.1895,349.2003	$C_{20}H_{29}O_5$-NicA
35	8.97	514.2432	514.2435	$[M+H]^+$	$C_{28}H_{36}O_8N$	-0.668	124.0395,295.1680,313.1802,331.1904,454.2220	$C_{20}H_{27}O_4$-NicA-AcA
36	9.10	635.2583	635.2599	$[M+H]^+$	$C_{34}H_{39}O_{10}N_2$	-2.553	124.0395,293.1537,311.1637,329.1761,347.1848,416.1854,434.1954	$C_{20}H_{25}O_4$-2NicA-AcA
							,452.2026,557.2280,617.2488	
37[a]	9.11	512.2271	512.2279	$[M+H]^+$	$C_{28}H_{34}O_8N$	-1.549	124.0395,293.1530,311.1633,329.1744,416.1857,434.1950,452.2062	$C_{20}H_{25}O_4$-NicA-AcA
38	9.12	574.2644	574.2647	$[M+H]^+$	$C_{30}H_{40}O_{10}N$	-0.475	124.0395,295.1688,313.1799,436.2122,454.2204	$C_{20}H_{27}O_4$-NicA-2AcA
39	9.19	575.2385	575.2388	$[M+H]^+$	$C_{32}H_{35}O_8N_2$	-0.508	124.0395,311.1646,434.1973,452.2065	$C_{20}H_{25}O_4$-2NicA
40[a]	9.21	458.2531	458.2532	$[M+H]^+$	$C_{26}H_{36}O_6N$	-1.340	124.0395,281.1894,299.2001	$C_{20}H_{31}O_4$-NicA
41	9.22	572.2480	572.2490	$[M+H]^+$	$C_{30}H_{38}O_{10}N$	-1.787	124.0395,293.1528,311.1632,329.1733,452.0341,470.2144,530.2368	$C_{20}H_{25}O_4$-NicA-2AcA
42	9.28	530.2377	530.2385	$[M+H]^+$	$C_{28}H_{36}O_9N$	-1.430	124.0394,329.1745,470.2209	$C_{20}H_{27}O_5$-NicA-AcA

43	9.30	514.2423	514.2435	$[M+H]^+$	$C_{28}H_{36}O_8N$	-2.418	124.0394,295.1696,313.1791,454.2220	$C_{20}H_{27}O_4$-NicA-AcA
44	9.30	577.2536	577.2544	$[M+H]^+$	$C_{32}H_{37}O_8N_2$	-1.460	124.0395,295.1691,313.1785,331.1902,454.2234	$C_{20}H_{27}O_4$-2NicA
45	9.30	592.2534	592.2541	$[M+H]^+$	$C_{33}H_{38}O_9N$	-1.196	124.0394,105.0338,293.1535,311.1626,329.1740, 347.1853,470.2172	$C_{20}H_{27}O_5$-NicA-BzA
46[a]	9.31	570.2320	570.2333	$[M+H]^+$	$C_{30}H_{36}O_{10}N$	-2.407	124.0394,291.1379,309.1478,327.1582,345.1689,450.1906,468.2010 ,510.2166,528.2217	$C_{20}H_{23}O_4$-NicA-2AcA
47	9.39	514.2428	514.2435	$[M+H]^+$	$C_{28}H_{36}O_8N$	-1.446	124.0395,295.1682,313.1798,331.1883,436.2110,454.2220	$C_{20}H_{27}O_4$-NicA-AcA
48	9.41	572.2475	572.2490	$[M+H]^+$	$C_{30}H_{38}O_{10}N$	-2.661	124.0395,311.1638,329.1744,454.2202,512.2298	$C_{20}H_{25}O_4$-NicA-2AcA
49[a]	9.42	528.2217	528.2228	$[M+H]^+$	$C_{28}H_{34}O_9N$	-2.098	124.0395,309.1480,327.1587,345.1680,450.1889,468.2019	$C_{20}H_{25}O_5$-NicA-AcA
50	9.43	470.2180	470.2173	$[M+H]^+$	$C_{26}H_{32}O_7N$	1.428	124.0394,311.1641,329.1740	$C_{20}H_{27}O_5$-NicA
51	9.50	544.2523	544.2541	$[M+H]^+$	$C_{29}H_{38}O_9N$	-3.322	124.0394,293.1530,311.1635,329.1738,434.1978,452.2055	$C_{20}H_{25}O_4$-NicA-X1
52	9.53	530.2371	530.2385	$[M+H]^+$	$C_{28}H_{36}O_9N$	-2.561	124.0394,293.1536,311.1685,329.1740,347.1837,434.1976,452.2048 ,470.2163,512.2249	$C_{20}H_{27}O_5$-NicA-AcA
53	9.55	574.2430	574.2435	$[M+H]^+$	$C_{33}H_{36}O_8N$	-0.946	124.0395,105.0338,293.1538,311.1635,329.1751,434.1960,452.2114	$C_{20}H_{25}O_4$-NicA-BzA
54	9.58	514.2429	514.2435	$[M+H]^+$	$C_{28}H_{36}O_8N$	-1.251	124.0393,295.1695,313.1790,436.2133,454.2233,496.2314	$C_{20}H_{27}O_4$-NicA-AcA
55	9.64	456.2368	456.2381	$[M+H]^+$	$C_{26}H_{34}O_6N$	-2.771	124.0395,297.1843,315.1950	$C_{20}H_{29}O_4$-NicA
56[a]	9.65	528.2217	528.2228	$[M+H]^+$	$C_{28}H_{34}O_9N$	-2.098	124.0394,309.1482,327.1602,345.1692,510.2106	$C_{20}H_{25}O_5$-NicA-AcA
57	9.66	544.2891	544.2905	$[M+H]^+$	$C_{30}H_{42}O_8N$	-2.561	124.0394,265.1945,283.2052,301.2157,406.2371,424.2474,484.2690	$C_{20}H_{29}O_2$-NicA-2AcA
58	9.68	510.2109	510.2122	$[M+H]^+$	$C_{28}H_{32}O_8N$	-2.633	124.0395,309.1475,327.1591,450.1902	$C_{20}H_{23}O_4$-NicA-AcA
59	9.73	574.2430	574.2435	$[M+H]^+$	$C_{33}H_{36}O_8N$	-0.946	124.0395,105.0338,293.1548,311.1630,434.1968,452.2056	$C_{20}H_{25}O_4$-NicA-BzA
60	9.75	544.2524	544.2541	$[M+H]^+$	$C_{29}H_{38}O_9N$	-3.139	124.0395,293.1530,311.1634,329.1756, 434.1953,452.2102,512.2271	$C_{20}H_{25}O_4$-NicA-X1
61	9.78	514.2431	514.2435	$[M+H]^+$	$C_{28}H_{36}O_8N$	-0.862	124.0394,295.1690,313.1791,331.1900,454.2212	$C_{20}H_{27}O_4$-NicA-AcA
62	9.79	558.2692	558.2698	$[M+H]^+$	$C_{30}H_{40}O_9N$	-1.000	124.0394,293.1533,311.1630,329.1740, 434.1961,452.2065,470.2178	$C_{20}H_{27}O_5$-NicA-A
63	9.81	572.2263	572.2279	$[M+H]^+$	$C_{33}H_{34}O_8N$	-2.785	124.0394,105.0338,291.1373,309.1480,327.1590,450.1890	$C_{20}H_{23}O_4$-NicA-BzA

64[a]	9.88	458.2176	458.2173	$[M+H]^+$	$C_{25}H_{32}O_7N$	0.592	124.0395,257.1543,275.1641,398.1948	$C_{17}H_{23}O_3$-NicA-AcA
65	9.89	530.2372	530.2385	$[M+H]^+$	$C_{28}H_{36}O_9N$	-2.373	124.0395,329.1721	$C_{20}H_{27}O_5$-NicA-AcA
66	9.90	454.2214	454.2224	$[M+H]^+$	$C_{26}H_{32}O_6N$	-2.233	124.0395	$C_{20}H_{27}O_4$-NicA
67	9.92	572.2475	572.2490	$[M+H]^+$	$C_{30}H_{38}O_{10}N$	-2.661	124.0394,311.1636,329.1737,470.2213,530.2401	$C_{20}H_{25}O_4$-NicA-2AcA
68[a]	9.96	458.2527	458.2537	$[M+H]^+$	$C_{26}H_{36}O_6N$	-2.213	124.0394,299.2006,317.2109,335.2209	$C_{20}H_{31}O_4$-NicA
69	10.00	558.2684	558.2698	$[M+H]^+$	$C_{30}H_{40}O_9N$	-2.433	124.0395,279.1742,297.1845,315.1956,420.2170,438.2278	$C_{20}H_{27}O_3$-NicA-2AcA
70	10.06	593.2491	593.2494	$[M+H]^+$	$C_{32}H_{37}O_9N_2$	-0.433	124.0395,293.1530, 311.1636,329.1743,347.1847,452.2068,470.2170	$C_{20}H_{27}O_5$-2NicA
71	10.07	572.2283	572.2279	$[M+H]^+$	$C_{33}H_{34}O_8N$	0.711	124.0395,105.0339, 291.1371,309.1483,327.1582,450.1881,512.2264	$C_{20}H_{23}O_4$-NicA-BzA
72	10.07	635.2591	635.2599	$[M+H]^+$	$C_{34}H_{39}O_{10}N_2$	-1.294	124.0394,293.1529,311.1631,329.1749,347.1846,434.1949,452.2057 ,512.2274,575.2385,617.2477	$C_{20}H_{25}O_4$-2NicA-AcA
73[a]	10.08	610.2648	610.2647	$[M+H]^+$	$C_{33}H_{40}O_{10}N$	0.208	124.0395,309.1480,327.1579,450.1906,550.2419,568.2535	$C_{20}H_{23}O_4$-NicA-C
74[a]	10.09	546.3040	546.3061	$[M+H]^+$	$C_{30}H_{44}O_8N$	-3.924	124.0395,267.2100,285.2209,303.2312,408.2528,426.2633,486.2843	$C_{20}H_{31}O_2$-NicA-2AcA
75[a]	10.09	502.2784	502.2799	$[M+H]^+$	$C_{28}H_{40}O_7N$	-3.044	124.0394,283.2054,301.2157,424.2480	$C_{20}H_{31}O_3$-NicA-AcA
76[a]	10.10	648.2422	648.2439	$[M+H]^+$	$C_{35}H_{38}O_{11}N$	-2.680	105.0338,124.0395,138.0549,343.1530,466.1851,588.2217	$C_{20}H_{23}O_5$-NicA-BzA-AcA
77	10.12	577.2544	577.2544	$[M+H]^+$	$C_{32}H_{37}O_8N_2$	-0.074	124.0395,295.1666,313.1804,331.1906,454.2212	$C_{20}H_{27}O_4$-2NicA
78[a]	10.13	528.2224	528.2228	$[M+H]^+$	$C_{28}H_{34}O_9N$	-0.772	124.0395,309.1462,327.1592,345.1681,468.1989	$C_{20}H_{25}O_5$-NicA-AcA
79[a]	10.15	412.2116	412.2118	$[M+H]^+$	$C_{24}H_{30}O_5N$	-0.605	124.0395,253.1582,271.1692	$C_{18}H_{25}O_3$-NicA
80	10.15	574.2429	574.2435	$[M+H]^+$	$C_{33}H_{36}O_8N$	-1.121	124.0395,105.0338,293.1534,311.1629,434.1969,452.2045,	$C_{20}H_{25}O_4$-NicA-BzA
81	10.19	514.2430	514.2435	$[M+H]^+$	$C_{28}H_{36}O_8N$	-1.057	124.0395, 295.1696,313.1795,331.1909,436.2114,454.2208	$C_{20}H_{27}O_4$-NicA-AcA
82	10.24	454.2220	454.2224	$[M+H]^+$	$C_{26}H_{32}O_6N$	-0.912	124.0394,313.1800,331.1915	$C_{20}H_{27}O_4$-NicA
83[a]	10.24	528.2214	528.2228	$[M+H]^+$	$C_{28}H_{34}O_9N$	-2.668	124.0394	$C_{20}H_{25}O_5$-NicA-AcA
84[a]	10.25	588.2786	588.2803	$[M+H]^+$	$C_{31}H_{42}O_{10}N$	-2.929	124.0395,295.1690,313.1794,331.1895,454.2217	$C_{20}H_{29}O_5$- NicA-B
85[a]	10.26	438.2262	438.2275	$[M+H]^+$	$C_{26}H_{32}O_5N$	-2.966	124.0394	$C_{20}H_{27}O_3$-NicA
86	10.34	558.2685	558.2698	$[M+H]^+$	$C_{30}H_{40}O_9N$	-2.254	124.0394,293.1532,311.1641,434.1968	$C_{20}H_{27}O_5$-NicA-A

87[a]	10.34	566.2383	566.2385	$[M+H]^+$	$C_{31}H_{36}O_9N$	-0.279	124.0395,309.1476,327.1574,432.1758,450.1901,468.2019	$C_{20}H_{23}O_4$-NicA-B
88	10.35	574.2435	574.2435	$[M+H]^+$	$C_{33}H_{36}O_8N$	-0.076	124.0395,105.0338,293.1538,311.1636,434.1957	$C_{20}H_{25}O_4$-NicA-BzA
89	10.36	470.2170	470.2173	$[M+H]^+$	$C_{26}H_{32}O_7N$	-0.699	124.0395,311.1643,329.1731	$C_{20}H_{27}O_5$-NicA
90	10.39	440.2424	440.2431	$[M+H]^+$	$C_{26}H_{34}O_5N$	-1.703	124.0395,299.2011,317.2101	$C_{20}H_{29}O_3$-NicA
91	10.41	514.2432	514.2435	$[M+H]^+$	$C_{28}H_{36}O_8N$	-0.668	124.0394,295.1686,313.1802,331.1903,454.2216	$C_{20}H_{27}O_4$-NicA-AcA
92	10.43	456.2372	456.2381	$[M+H]^+$	$C_{26}H_{34}O_6N$	-1.894	124.0395,297.1849,315.1951	$C_{20}H_{29}O_4$-NicA
93	10.45	575.2389	575.2388	$[M+H]^+$	$C_{32}H_{35}O_8N_2$	0.183	124.0395,293.1538,311.1636,329.1742,434.1954,452.2067	$C_{20}H_{25}O_4$-2NicA
94	10.45	572.2253	572.2279	$[M+H]^+$	$C_{33}H_{34}O_8N$	-2.435	124.0395,105.0338,291.1372,309.1482,327.1595,450.1901	$C_{20}H_{23}O_4$-NicA-BzA
95	10.47	530.2382	530.2385	$[M+H]^+$	$C_{28}H_{36}O_9N$	-0.478	124.0395,311.1646,329.1758	$C_{20}H_{27}O_5$-NicA-AcA
96	10.49	556.2526	556.2541	$[M+H]^+$	$C_{30}H_{38}O_9N$	-2.711	124.0395,295.1681,313.1786,436.0740	$C_{20}H_{25}O_3$-NicA-2AcA
97	10.54	577.2537	577.2544	$[M+H]^+$	$C_{32}H_{37}O_8N_2$	-1.286	124.0394,295.1691,313.1792,436.2114,454.2213	$C_{20}H_{27}O_4$-2NicA
98[a]	10.56	412.2116	412.2118	$[M+H]^+$	$C_{24}H_{30}O_5N$	-0.605	124.0394,293.2104	$C_{18}H_{25}O_3$-NicA
99	10.56	530.2379	530.2385	$[M+H]^+$	$C_{28}H_{36}O_9N$	-1.053	124.0395,329.1719,347.1842	$C_{20}H_{27}O_5$-NicA-AcA
100[a]	10.57	588.2793	588.2803	$[M+H]^+$	$C_{31}H_{42}O_{10}N$	-1.793	124.0395,345.2056,327.1950,309.1852,450.2250,468.2372,528.2568	$C_{21}H_{29}O_4$- NicA-2AcA
101	10.61	592.2536	592.2541	$[M+H]^+$	$C_{33}H_{38}O_9N$	-0.858	124.0395,105.0338,311.1694,329.1742,452.2067,470.2172	$C_{20}H_{27}O_5$-NicA-BzA
102	10.66	552.2590	552.2592	$[M+H]^+$	$C_{31}H_{38}O_8N$	-0.350	124.0395,295.1689,313.1795,331.1898,418.2014,436.2094,454.2215	$C_{20}H_{25}O_3$- NicA-B
103 [a]	10.67	570.2689	570.2698	$[M+H]^+$	$C_{31}H_{40}O_9N$	-1.505	124.0395,295.1688,313.1801,331.1896,418.2024,436.2113,534.2487 ,552.2591	$C_{20}H_{27}O_4$-NicA-B
104 [a]	10.67	500.2642	500.2643	$[M+H]^+$	$C_{28}H_{38}O_7N$	-0.158	124.0394,281.1894,299.1999,317.2109,422.2328,440.2406	$C_{20}H_{29}O_3$- NicA-AcA
105	10.70	544.2521	544.2541	$[M+H]^+$	$C_{29}H_{38}O_9N$	-3.690	124.0394,293.1535,311.1632,329.1732,434.1937,452.2073	$C_{20}H_{25}O_4$-NicA-X1
106 [a]	10.71	512.2265	512.2279	$[M+H]^+$	$C_{28}H_{34}O_8N$	-2.720	124.0394,311.1630	$C_{20}H_{25}O_4$-NicA-AcA
107	10.72	595.2632	595.2650	$[M+H]^+$	$C_{32}H_{39}O_9N_2$	-3.306	124.0395,271.1689,394.2001,472.2322,517.2340,577.2510	$C_{18}H_{25}O_3$- 2NicA-AcA
108	10.74	556.2537	556.2541	$[M+H]^+$	$C_{30}H_{38}O_9N$	-0.374	124.0395,295.1688,313.1796,331.1907,436.0633,496.2327	$C_{20}H_{25}O_3$-NicA-2AcA
109	10.76	575.2374	575.2388	$[M+H]^+$	$C_{32}H_{35}O_8N_2$	-2.421	124.0395,311.1633,329.1743,452.2060	$C_{20}H_{25}O_4$-2NicA
110 [a]	10.76	614.2957	614.2960	$[M+H]^+$	$C_{33}H_{44}O_{10}N$	-0.444	124.0394,295.1688,313.1794,331.1897,418.2003,436.2106,454.2212 ,536.2637,554.2755,578.2749,596.2849	$C_{20}H_{27}O_4$-NicA-C

111 [a]	10.78	570.2686	570.2698	$[M+H]^+$	$C_{31}H_{40}O_9N$	-2.031	124.0395,313.1783	$C_{20}H_{27}O_4$-NicA-B
112	10.78	596.2852	596.2854	$[M+H]^+$	$C_{33}H_{42}O_9N$	-0.349	124.0394,295.1687,313.1789,331.1918,418.2024,436.2113,454.2224 ,536.2641,554.2774,578.2745,596.2852	$C_{20}H_{25}O_3$-NicA-C
113	10.81	514.2427	514.2435	$[M+H]^+$	$C_{28}H_{36}O_8N$	-1.640	124.0395,295.1687,313.1796,331.1796,436.2141,496.2346	$C_{20}H_{27}O_4$-NicA-AcA
114	10.85	633.2434	633.2443	$[M+H]^+$	$C_{34}H_{37}O_{10}N_2$	-1.377	124.0395,291.1361,309.1483,327.1587,432.1807,450.1895,492.2028 ,510.2094,555.2115,573.2230	$C_{20}H_{23}O_4$-2NicA-AcA
115	10.85	572.2468	572.2490	$[M+H]^+$	$C_{30}H_{38}O_{10}N$	-3.884	124.0395,293.1535,311.1634,329.1742,434.2003,452.2078, 512.2267	$C_{20}H_{25}O_4$-NicA-2AcA
116	10.86	592.2536	592.2541	$[M+H]^+$	$C_{33}H_{38}O_9N$	-0.858	124.0395,105.0338,311.1631,329.1745,434.1955,452.2055,470.2160	$C_{20}H_{27}O_5$-NicA-BzA
117	10.86	554.2372	554.2385	$[M+H]^+$	$C_{30}H_{36}O_9N$	-2.270	124.0394,293.1529,311.1635,329.1742,434.1972, 452.2051,512.2227	$C_{20}H_{23}O_3$-NicA-2AcA
118[a]	10.89	512.2238	512.2279	$[M+H]^+$	$C_{28}H_{34}O_8N$	-7.991	124.0394,293.1534,311.1642,329.1742,347.1835,452.2102	$C_{20}H_{25}O_4$-NicA-AcA
119	10.91	514.2429	514.2435	$[M+H]^+$	$C_{28}H_{36}O_8N$	-1.251	124.0394,295.1692,313.1790,331.1906,454.2190	$C_{20}H_{27}O_4$-NicA-AcA
120[a]	10.91	616.2518	616.2541	$[M+H]^+$	$C_{35}H_{38}O_9N$	-3.745	124.0394,105.0338,293.1528,311.1636,329.1748,434.1940,556.2518	$C_{20}H_{23}O_3$-NicA-BzA-AcA
121[a]	10.93	570.2673	570.2698	$[M+H]^+$	$C_{31}H_{40}O_9N$	-4.311	124.0395,295.1687,313.1798,331.1896,418.2024,552.2589	$C_{20}H_{27}O_4$-NicA-B
122	10.93	635.2588	635.2599	$[M+H]^+$	$C_{34}H_{39}O_{10}N_2$	-1.766	124.0394,293.1528,311.1636,329.1741,347.1877,434.1957,452.2058 ,512.2209,557.2263,575.2375,617.2477	$C_{20}H_{25}O_4$-2NicA-AcA
123	10.94	559.2436	559.2439	$[M+H]^+$	$C_{32}H_{35}O_7N_2$	-0.497	124.0395,295.1690,313.1794,436.2113	$C_{20}H_{25}O_3$-2NicA
124	10.95	440.2442	440.2431	$[M+H]^+$	$C_{26}H_{34}O_5N$	2.386	124.0395,299.2002	$C_{20}H_{29}O_3$-NicA
125	10.96	574.2418	574.2435	$[M+H]^+$	$C_{33}H_{36}O_8N$	-3.036	124.0395,105.0338,293.1525,311.1635,329.1747,416.1854, 434.1943	$C_{20}H_{25}O_4$-NicA-BzA
126	10.99	592.2531	592.2541	$[M+H]^+$	$C_{33}H_{38}O_9N$	-1.702	124.0395,105.0338,311.1644,329.1747,434.1974,452.2062,470.2170	$C_{20}H_{27}O_5$-NicA-BzA
127	11.02	514.2432	514.2435	$[M+H]^+$	$C_{28}H_{36}O_8N$	-0.668	124.0395,313.1811,331.1909,373.1990	$C_{20}H_{27}O_4$-NicA-AcA
128	11.02	619.2639	619.2650	$[M+H]^+$	$C_{34}H_{39}O_9N_2$	-1.768	124.0395,295.1689,313.1811,436.2144,454.2220,496.2325,577.2540	$C_{20}H_{25}O_3$-2NicA-AcA
129	11.06	574.2430	574.2435	$[M+H]^+$	$C_{33}H_{36}O_8N$	-0.946	124.0394,105.0338,311.1633,329.1749,452.2061,434.1943	$C_{20}H_{25}O_4$-NicA-BzA
130[a]	11.09	588.2780	588.2803	$[M+H]^+$	$C_{31}H_{42}O_{10}N$	-0.039	124.0395,345.2052,327.1954,309.1844,450.2254,468.2385,528.2568	$C_{21}H_{29}O_4$-NicA-2AcA

131	11.14	440.2435	440.2431	$[M+H]^+$	$C_{26}H_{34}O_5N$	0.796	124.0395,299.2004	$C_{20}H_{29}O_3$-NicA
132	11.16	557.2275	557.2282	$[M+H]^+$	$C_{32}H_{33}O_7N_2$	-1.306	124.0395,293.1531,311.1635,434.1954	$C_{20}H_{23}O_3$-2NicA
133	11.16	576.2582	576.2592	$[M+H]^+$	$C_{33}H_{38}O_8N$	-1.724	124.0395,105.0338,295.1687,313.1793,331.1898,558.2471	$C_{20}H_{27}O_4$-NicA-BzA
134	11.18	530.2750	530.2748	$[M+H]^+$	$C_{29}H_{40}O_8N$	0.259	124.03945,297.1844,315.1948,438.2264,470.2530	$C_{20}H_{27}O_3$-NicA-X1
135 [a]	11.20	570.2689	570.2698	$[M+H]^+$	$C_{31}H_{40}O_9N$	-1.505	124.0395,295.1689,313.1795,331.1915,418.2010,436.2118,454.2238 ,552.2576,534.2488	$C_{20}H_{27}O_4$-NicA-B
136	11.20	682.2751	682.2759	$[M+H]^+$	$C_{38}H_{40}O_9N_3$	-1.182	124.0394,295.1689,313.1791,436.2100,559.2431	$C_{20}H_{25}O_3$-3NicA
137	11.23	456.2387	456.2381	$[M+H]^+$	$C_{26}H_{34}O_6N$	1.394	124.0395,297.1850,315.1955,438.2239	$C_{20}H_{29}O_4$-NicA
138	11.27	556.2526	556.2541	$[M+H]^+$	$C_{30}H_{38}O_9N$	-2.711	124.0394,295.1689,313.1788,514.2420	$C_{20}H_{25}O_3$-NicA-2AcA
139 [a]	11.30	570.2674	570.2698	$[M+H]^+$	$C_{31}H_{40}O_9N$	-4.135	124.0395,295.1687,313.1798,436.2122,552.2586	$C_{20}H_{27}O_4$-NicA-B
140	11.32	496.2322	496.2330	$[M+H]^+$	$C_{28}H_{34}O_7N$	-1.569	124.0394,295.1688,313.1796,331.1901,436.2120	$C_{20}H_{25}O_3$-NicA-AcA
141	11.33	514.2413	514.2435	$[M+H]^+$	$C_{28}H_{36}O_8N$	-4.363	124.0395,295.1677,313.1793,331.1887,468.1963,514.2435	$C_{20}H_{27}O_4$-NicA-AcA
142	11.34	456.2374	456.2381	$[M+H]^+$	$C_{26}H_{34}O_6N$	-1.456	124.0394,297.1837,315.1952,333.2046	$C_{20}H_{29}O_4$-NicA
143	11.34	680.2596	680.2603	$[M+H]^+$	$C_{38}H_{38}O_9N_3$	-0.965	124.0395,293.1523,311.1632,434.1954,557.2277,	$C_{20}H_{23}O_3$-3NicA
144	11.35	576.2587	576.2592	$[M+H]^+$	$C_{33}H_{38}O_8N$	-0.856	124.0395,105.0338,295.1694,313.1796,331.1902,454.2269	$C_{20}H_{27}O_4$-NicA-BzA
145	11.38	568.2531	568.2541	$[M+H]^+$	$C_{31}H_{38}O_9N$	-1.774	124.0395,293.1535,311.1637,329.1745, 452.2070,532.2409,550.2794	$C_{20}H_{25}O_4$-NicA-B
146[a]	11.39	452.2063	452.2068	$[M+H]^+$	$C_{26}H_{30}O_6N$	-1.026	124.0395	$C_{20}H_{25}O_4$-NicA
147	11.44	574.2433	574.2435	$[M+H]^+$	$C_{33}H_{36}O_8N$	-0.424	124.0394,105.0338,311.1633	$C_{20}H_{25}O_4$-NicA-BzA
148	11.47	559.2426	559.2439	$[M+H]^+$	$C_{32}H_{35}O_7N_2$	-2.285	124.0394,295.1680,313.1785,436.2110	$C_{20}H_{25}O_3$-2NicA
149	11.47	556.2537	556.2541	$[M+H]^+$	$C_{30}H_{38}O_9N$	-0.734	124.0394,295.1698,313.1795,331.1895,454.2236,496.2310,514.2515	$C_{20}H_{25}O_3$-NicA-2AcA
150[a]	11.49	570.2697	570.2698	$[M+H]^+$	$C_{31}H_{40}O_9N$	-0.102	124.0395,295.1690,313.1787,552.2604	$C_{20}H_{27}O_4$-NicA-B
151[a]	11.49	628.2726	628.2752	$[M+H]^+$	$C_{33}H_{42}O_{11}N$	-4.918	124.0394,329.1746,512.2247	$C_{20}H_{25}O_4$-NicA-AcA-B
152[a]	11.50	442.2580	442.2588	$[M+H]^+$	$C_{26}H_{36}O_5N$	-1.808	124.0394,283.2051,301.2158,424.2476	$C_{20}H_{31}O_3$-NicA
153	11.54	634.2630	634.2647	$[M+H]^+$	$C_{35}H_{40}O_{10}N$	-2.637	124.0394,105.0338,293.1523,311.1631,329.1740,347.1838,434.1932 ,512.2233,556.2323,616.2516	$C_{20}H_{25}O_4$-NicA-BzA-AcA

154	11.56	554.2369	554.2385	$[M+H]^+$	$C_{30}H_{36}O_9N$	-2.811	124.0395,293.1526,311.1631,329.1736,434.1957,452.2038,494.2216 ,512.2233	$C_{20}H_{23}O_3$-NicA-2AcA
155[a]	11.57	438.2264	438.2275	$[M+H]^+$	$C_{26}H_{32}O_5N$	-2.509	124.0395,297.1852,315.1952	$C_{20}H_{27}O_3$-NicA
156[a]	11.60	542.2740	542.2748	$[M+H]^+$	$C_{30}H_{40}O_8N$	-1.556	124.0395,295.1685,313.1791,331.1895,436.2494,454.2194,482.2539	$C_{20}H_{27}O_4$-NicA-A
157	11.61	530.2753	530.2748	$[M+H]^+$	$C_{29}H_{40}O_8N$	0.861	124.0394,297.1844,315.1954,420.2166,438.2272	$C_{20}H_{27}O_3$-NicA-X1
158	11.61	561.2590	561.2595	$[M+H]^+$	$C_{32}H_{37}O_7N_2$	-0.941	124.0395,297.1843,315.1949,420.2156,438.2266,543.2510	$C_{20}H_{27}O_3$-2NicA
159[a]	11.65	534.2485	534.2486	$[M+H]^+$	$C_{31}H_{36}O_7N$	-0.129	124.0395,105.0338	$C_{18}H_{25}O_3$-NicA-BzA
160	11.71	682.2742	682.2759	$[M+H]^+$	$C_{38}H_{40}O_9N_3$	-2.501	124.0395,295.1689,313.1796,436.2127,559.2426	$C_{20}H_{25}O_3$-3NicA
161	11.72	619.2645	619.2650	$[M+H]^+$	$C_{34}H_{39}O_9N_2$	-0.819	124.0395,295.1691,313.1793,436.2106,454.2250,496.2325,559.2361	$C_{20}H_{25}O_3$-2NicA-AcA
162[a]	11.75	570.2687	570.2698	$[M+H]^+$	$C_{31}H_{40}O_9N$	-1.856	124.0395,295.1688,313.1793,331.1899,436.2109,454.3622,552.2585	$C_{20}H_{27}O_4$-NicA-B
163	11.76	554.2755	554.2748	$[M+H]^+$	$C_{31}H_{40}O_8N$	1.184	124.0395,295.1692,313.1795,331.1895,418.2042,436.2114,536.2663	$C_{20}H_{27}O_4$-NicA-X2
164[a]	11.80	498.2487	498.2486	$[M+H]^+$	$C_{28}H_{36}O_7N$	0.143	124.0395,297.1847,315.1953	$C_{20}H_{27}O_3$-NicA-AcA
165	11.81	576.2585	576.2592	$[M+H]^+$	$C_{33}H_{38}O_8N$	-1.204	124.0394,105.0338,295.1691,313.1790,331.1894,418.2014,436.2063 ,454.2219	$C_{20}H_{27}O_4$-NicA-BzA
166[a]	11.86	542.2740	542.2748	$[M+H]^+$	$C_{30}H_{40}O_8N$	-1.556	124.0394,313.1788,454.2215	$C_{20}H_{27}O_4$-NicA-A
167	11.90	556.2899	556.2905	$[M+H]^+$	$C_{31}H_{42}O_8N$	-1.067	124.0394,297.1841,315.1949,420.2153,438.2267,538.2813	$C_{20}H_{29}O_4$-NicA-X2
168	11.91	619.2645	619.2650	$[M+H]^+$	$C_{34}H_{39}O_9N_2$	-0.819	124.0395,295.1687,313.1794,418.2037,436.2116,454.2215,496.2312 ,559.2423	$C_{20}H_{25}O_3$-2NicA-AcA
169	11.91	576.2587	576.2592	$[M+H]^+$	$C_{33}H_{38}O_8N$	-0.856	124.0395,313.1801,331.1903	$C_{20}H_{27}O_4$-NicA-BzA
170[a]	11.92	570.2696	570.2698	$[M+H]^+$	$C_{31}H_{40}O_9N$	-0.227	124.0395,295.1685,313.1795,331.1896,454.2225,552.2587	$C_{20}H_{27}O_4$-NicA-B
171[a]	11.96	553.2421	553.2432	$[M+H]^+$	$C_{31}H_{37}O_9$	-2.005	105.0339,293.1532,311.1637,329.1741,433.2011	$C_{20}H_{23}O_3$-BzA-2AcA
172[a]	11.99	498.2477	498.2486	$[M+H]^+$	$C_{28}H_{36}O_7N$	-1.864	124.0394,297.1852,315.1945,438.2282	$C_{20}H_{27}O_3$-NicA-AcA
173	11.99	561.2573	561.2595	$[M+H]^+$	$C_{32}H_{37}O_7N_2$	-3.969	124.0395,297.1835,315.1960,438.2274	$C_{20}H_{27}O_3$-2NicA
174	12.03	556.2531	556.2541	$[M+H]^+$	$C_{30}H_{38}O_9N$	-1.812	124.0395,297.1852,315.1952,420. 438.2252	$C_{20}H_{29}O_4$-NicA-X2
175	12.05	496.2325	496.2330	$[M+H]^+$	$C_{28}H_{34}O_7N$	-0.965	124.0395,295.1688,313.1931,436.2136	$C_{20}H_{25}O_3$-NicA-AcA
176	12.07	552.2585	552.2592	$[M+H]^+$	$C_{31}H_{38}O_8N$	-1.256	124.0395,295.1689,313.1792,436.2115,454.2212	$C_{20}H_{25}O_3$-NicA-B

177	12.09	556.2526	556.2541	$[M+H]^+$	$C_{30}H_{38}O_9N$	-2.711	124.0394,297.1847,315.1945,420.2128,438.2284	$C_{20}H_{29}O_4$-NicA-X2
178	12.09	576.2587	576.2592	$[M+H]^+$	$C_{33}H_{38}O_8N$	-0.856	124.0394,295.1683,313.1785,331.1898,436.2094,454.2209	$C_{20}H_{27}O_4$-NicA-BzA
179	12.11	619.2645	619.2650	$[M+H]^+$	$C_{34}H_{39}O_9N_2$	-0.819	124.0395,295.1684,313.1794,436.2134,496.2330	$C_{20}H_{25}O_3$-2NicA-AcA
180[a]	12.13	598.2630	598.2647	$[M+H]^+$	$C_{32}H_{40}O_{10}N$	-2.796	124.0395,309.1477,327.1582,450.1894,510.2135,557.2551	$C_{20}H_{23}O_4$-NicA-AcA-A
181[a]	12.15	570.2692	570.2698	$[M+H]^+$	$C_{31}H_{40}O_9N$	-0.979	124.0395,295.1689,313.1787,331.1917,436.2118,552.2593	$C_{20}H_{27}O_4$-NicA-B
182[a]	12.15	614.2955	614.2960	$[M+H]^+$	$C_{33}H_{44}O_{10}N$	-0.770	124.0395,295.1687,313.1793,331.1900,418.2007,436.2120,454.2202 ,554.2720,572.2849,596.2850	$C_{20}H_{27}O_4$-NicA-C
183	12.20	576.2592	576.2592	$[M+H]^+$	$C_{33}H_{38}O_8N$	0.011	124.0394,295.1687,313.1795,331.1896,436.2100	$C_{20}H_{27}O_4$-NicA-BzA
184[a]	12.24	600.2794	600.2803	$[M+H]^+$	$C_{32}H_{42}O_{10}N$	-1.537	124.0394,293.1542,311.1636,329.1743,347.1843,434.2002,452.2059 ,470.2159,512.2280,540.2589,582.2725	$C_{20}H_{25}O_4$-NicA-AcA-A
185	12.24	556.2886	556.2905	$[M+H]^+$	$C_{31}H_{42}O_8N$	-3.404	124.0395,297.1845,315.1957,438.2238,538.2886	$C_{20}H_{29}O_4$-NicA-X2
186[a]	12.25	582.2695	582.2698	$[M+H]^+$	$C_{32}H_{40}O_9N$	-0.443	124.0394,293.1534,311.1637,329.1742,434.1963,452.2059,470.2188 ,512.2273,540.2569	$C_{20}H_{23}O_3$-NicA-AcA-A
187	12.26	561.2590	561.2595	$[M+H]^+$	$C_{32}H_{37}O_7N_2$	-0.941	124.0394,297.1843,315.1948,438.2270	$C_{20}H_{27}O_3$-2NicA
188	12.26	619.2642	619.2650	$[M+H]^+$	$C_{34}H_{39}O_9N_2$	-1.303	124.0395,295.1678,313.1794,436.2113,454.2178,496.2327,559.2434 ,601.2766	$C_{20}H_{25}O_3$-2NicA-AcA
189[a]	12.27	512.2269	512.2279	$[M+H]^+$	$C_{28}H_{34}O_8N$	-1.939	124.0394,293.1528,311.1635,329.1741,347.1864,452.2057,470.2157	$C_{20}H_{25}O_4$-NicA-AcA
190[a]	12.28	498.2482	498.2486	$[M+H]^+$	$C_{28}H_{36}O_7N$	-0.861	124.0395,297.1845,315.1948,420.2169,438.2280	$C_{20}H_{27}O_3$-NicA-AcA
191	12.29	544.2896	544.2905	$[M+H]^+$	$C_{30}H_{42}O_8N$	-1.642	124.0394,297.1851,315.1946,420.2163,438.2268	$C_{20}H_{27}O_3$-NicA-X3
192	12.30	584.2848	584.2854	$[M+H]^+$	$C_{32}H_{42}O_9N$	-1.041	124.0395,295.1685,313.1788,331.1883,454.2151,514.2390	$C_{20}H_{25}O_3$-NicA-AcA-A
193	12.32	524.2643	524.2643	$[M+H]^+$	$C_{30}H_{38}O_7N$	0.040	124.0395,295.1692,313.1789,331.1891,436.2119,454.2215	$C_{20}H_{25}O_3$-NicA-A
194	12.33	576.2589	576.2592	$[M+H]^+$	$C_{33}H_{38}O_8N$	-0.509	124.0394,295.1689,313.1795,331.1916,436.2103,454.2225,558.2589	$C_{20}H_{27}O_4$-NicA-BzA
195	12.34	530.2743	530.2748	$[M+H]^+$	$C_{29}H_{40}O_8N$	-1.025	124.0394,311.1997,329.2105,347.2211,470.2506,498.2465,438.2301 ,297.1843,315.1946	$C_{21}H_{31}O_4$-NicA-AcA
196[a]	12.37	594.2687	594.2698	$[M+H]^+$	$C_{33}H_{40}O_9N$	-1.781	124.0394,105.0338,289.1792,271.1687,253.1582,393.2070,516.2355	$C_{18}H_{25}O_3$-NicA-BzA-AcA
197	12.39	514.2434	514.2435	$[M+H]^+$	$C_{28}H_{36}O_8N$	-0.279	124.0395	$C_{20}H_{27}O_4$-NicA-AcA

198	12.39	559.2432	559.2439	$[M+H]^+$	$C_{32}H_{35}O_7N_2$	-1.212	124.0394,295.1687,313.1792,436.2111	$C_{20}H_{25}O_3$-2NicA
199[a]	12.42	545.2636	545.2646	$[M+H]^+$	$C_{32}H_{37}O_6N_2$	-1.858	124.0394,299.1998,422.2310	$C_{20}H_{27}O_2$-2NicA
200[a]	12.43	498.2410	498.2486	$[M+H]^+$	$C_{28}H_{36}O_7N$	-0.058	124.0394,297.1835,315.1947,438.2260	$C_{20}H_{27}O_3$-NicA-AcA
201	12.45	561.2591	561.2595	$[M+H]^+$	$C_{32}H_{37}O_7N_2$	-0.762	124.0394,297.1848,315.1950,438.2273	$C_{20}H_{27}O_3$-2NicA
202	12.47	554.2740	554.2748	$[M+H]^+$	$C_{31}H_{40}O_8N$	-1.522	124.0395,297.1848,315.1947,420.2173	$C_{20}H_{27}O_3$-NicA-B
203	12.51	559.2433	559.2439	$[M+H]^+$	$C_{32}H_{35}O_7N_2$	-1.033	124.0394,295.1686,313.1794,436.2119	$C_{20}H_{25}O_3$-2NicA
204	12.51	634.2642	634.2647	$[M+H]^+$	$C_{35}H_{40}O_{10}N$	-0.745	105.0338,124.0394,293.1533,311.1641,329.1745,347.1838,434.1962 ,452.2054,512.2277,574.2429,616.2516	$C_{20}H_{25}O_4$-NicA-BzA-AcA
205	12.52	574.2429	574.2435	$[M+H]^+$	$C_{33}H_{36}O_8N$	-1.121	124.0394,105.0337,293.1535,311.1642,329.1735,452.2045	$C_{20}H_{25}O_4$-NicA-BzA
206[a]	12.55	647.2940	647.2963	$[M+H]^+$	$C_{36}H_{43}O_9N_2$	-3.565	124.0394,295.1680,313.1792,436.2093,454.2213,524.2637,577.2538	$C_{20}H_{25}O_3$-2NicA-A
207	12.56	556.2532	556.2541	$[M+H]^+$	$C_{30}H_{38}O_9N$	-1.633	124.0395,295.1688,313.1797,331.1874,436.2136	$C_{20}H_{25}O_3$-NicA-2AcA
208[a]	12.58	570.2689	570.2698	$[M+H]^+$	$C_{31}H_{40}O_9N$	-1.505	124.0395,295.1685,313.1795,454.2220,552.2614	$C_{20}H_{27}O_4$-NicA-B
209	12.60	576.2587	576.2592	$[M+H]^+$	$C_{33}H_{38}O_8N$	-0.856	124.0395,295.1696,313.1791,331.1901,436.2132,454.2244,558.2469 ,105.0338	$C_{20}H_{27}O_4$-NicA-BzA
210	12.63	552.2587	552.2592	$[M+H]^+$	$C_{31}H_{38}O_8N$	-0.894	124.0394,295.1689,313.1794,331.1901,436.2108,454.2246	$C_{20}H_{25}O_3$-NicA-B
211[a]	12.66	511.2313	511.2326	$[M+H]^+$	$C_{29}H_{35}O_8$	-2.630	105.0338,293.1533,311.1641,329.1742,347.1866	$C_{20}H_{25}O_4$-BzA-AcA
212	12.67	496.2316	496.2330	$[M+H]^+$	$C_{28}H_{34}O_7N$	-2.779	124.0394,295.1693,313.1791,331.1902,436.2118,454.2221	$C_{20}H_{25}O_3$-NicA-AcA
213[a]	12.67	632.2482	632.2490	$[M+H]^+$	$C_{35}H_{38}O_{10}N$	-1.301	124.0395,105.0338,309.1479,327.1589,572.2279	$C_{20}H_{23}O_4$-NicA-BzA-AcA
214	12.68	556.2533	556.2541	$[M+H]^+$	$C_{30}H_{38}O_9N$	-1.453	124.0394,295.1695,313.1792,331.1903,373.2005,496.2324	$C_{20}H_{25}O_3$-NicA-2AcA
215[a]	12.70	540.2956	540.2956	$[M+H]^+$	$C_{31}H_{42}O_7N$	0.039	124.0395,281.1890,299.2003,422.2320	$C_{20}H_{29}O_3$-NicA-X2
216	12.71	584.2848	584.2854	$[M+H]^+$	$C_{32}H_{42}O_9N$	-1.041	124.0395,295.1687,313.1796,331.1904,454.2234,496.2337	$C_{20}H_{25}O_3$-NicA-AcA-A
217	12.75	544.2892	544.2905	$[M+H]^+$	$C_{30}H_{42}O_8N$	-2.377	124.0394,297.1845,315.1955,420.2128,438.2273	$C_{20}H_{27}O_3$-NicA-X3
218	12.76	634.2640	634.2647	$[M+H]^+$	$C_{35}H_{40}O_{10}N$	-1.061	105.0338,124.0394,293.1533,311.1634,329.1740,347.1838,433.2009 ,452.2039,556.2287,574.2449,616.2546	$C_{20}H_{25}O_4$-NicA-BzA-AcA
219	12.79	524.2640	524.2643	$[M+H]^+$	$C_{30}H_{38}O_7N$	-0.532	124.0395,295.1681,313.1795,331.1898,436.2116,454.2226	$C_{20}H_{25}O_3$-NicA-A
220	12.79	493.2206	493.2221	$[M+H]^+$	$C_{29}H_{33}O_7$	-3.000	105.0339,293.1530,311.1619	$C_{20}H_{23}O_3$-BzA-AcA

221[a]	12.79	512.2264	512.2279	$[M+H]^+$	$C_{28}H_{34}O_8N$	-2.916	124.0395,293.1556,311.1635,329.1746,452.2057,470.2195	$C_{20}H_{25}O_4$-NicA-AcA
222	12.80	576.2589	576.2592	$[M+H]^+$	$C_{33}H_{38}O_8N$	-0.509	124.0395,313.1791,454.2181,105.0338	$C_{20}H_{27}O_4$-NicA-BzA
223	12.81	470.2169	470.2173	$[M+H]^+$	$C_{26}H_{32}O_7N$	-0.912	124.0394,293.1543,311.1645,329.1742,347.1848,452.2090	$C_{20}H_{27}O_5$-NicA
224	12.83	596.2834	596.2854	$[M+H]^+$	$C_{33}H_{42}O_9N$	-3.368	124.0394,295.1687,313.1794,418.2000,436.2109,454.2209,554.2738 ,578.2789	$C_{20}H_{25}O_3$-NicA-C
225[a]	12.84	672.3004	672.3015	$[M+H]^+$	$C_{35}H_{46}O_{12}N$	-1.565	124.0395,293.1531,311.1637,329.1743,434.1945,452.2062,494.2160 ,512.2271,594.2693,612.2816,654.2895	$C_{20}H_{25}O_4$-NicA-AcA-C
226[a]	12.87	647.2957	647.2963	$[M+H]^+$	$C_{36}H_{43}O_9N_2$	-0.938	124.0394,295.1690,313.1792,436.2098,454.2270,559.2423	$C_{20}H_{25}O_3$-2NicA-A
227	12.91	618.2688	618.2698	$[M+H]^+$	$C_{35}H_{40}O_9N$	-1.550	124.0394,105.0338,295.1687,313.1792,436.2122,558.2483	$C_{20}H_{25}O_3$-NicA-BzA-AcA
228	12.92	558.2485	558.2486	$[M+H]^+$	$C_{33}H_{36}O_7N$	-0.231	124.0395,105.0338,295.1683,313.1800,436.2124	$C_{20}H_{25}O_3$-NicA-BzA
229[a]	12.96	452.2065	452.2068	$[M+H]^+$	$C_{26}H_{30}O_6N$	-0.584	124.0395,293.1534,311.1651,329.1743	$C_{20}H_{25}O_4$-NicA
230	12.96	470.2163	470.2173	$[M+H]^+$	$C_{26}H_{32}O_7N$	-2.188	124.0394,293.1538,329.1741,347.1848,452.2054	$C_{20}H_{27}O_5$-NicA
231[a]	12.98	570.2672	570.2698	$[M+H]^+$	$C_{31}H_{40}O_9N$	-4.486	124.0395,295.1687,313.1794,331.1898,436.2110,454.2285,552.2607	$C_{20}H_{27}O_4$-NicA-B
232	12.99	592.2540	592.2541	$[M+H]^+$	$C_{33}H_{38}O_9N$	-0.183	124.0394,293.1530,329.1744,452.2067	$C_{20}H_{27}O_5$-NicA-BzA
233[a]	13.01	482.2531	482.2537	$[M+H]^+$	$C_{28}H_{36}O_6N$	-1.274	124.0395,299.2002,317.2105	$C_{20}H_{27}O_2$-NicA-AcA
234[a]	13.03	659.2957	659.2963	$[M+H]^+$	$C_{37}H_{43}O_9N_2$	-0.921	124.0394,295.1683,313.1790,418.1998,436.2098,518.2516,536.2629 ,641.2849	$C_{20}H_{25}O_3$-2NicA-X2
235[a]	13.07	498.2480	498.2486	$[M+H]^+$	$C_{28}H_{36}O_7N$	-1.262	124.0394,297.1841,315.1951,438.2279	$C_{20}H_{27}O_3$-NicA-AcA
236	13.07	556.2319	556.2330	$[M+H]^+$	$C_{33}H_{34}O_7N$	-1.940	124.0394,105.0338,293.1527,311.1636,434.1960	$C_{20}H_{23}O_3$-NicA-BzA
237	13.10	576.2601	576.2592	$[M+H]^+$	$C_{33}H_{38}O_8N$	1.573	124.0394,105.0338,295.1689,313.1793,331.1890,454.2237	$C_{20}H_{27}O_4$-NicA-BzA
238[a]	13.10	650.2589	650.2596	$[M+H]^+$	$C_{35}H_{40}O_{11}N$	-1.057	124.0395,293.1529,311.1635,329.1743,347.1844,452.2058,470.2174 ,512.2281,530.2382,572.2325,590.2366	$C_{20}H_{25}O_5$-NicA-BzA-AcA
239[a]	13.11	436.2110	436.2118	$[M+H]^+$	$C_{26}H_{30}O_5N$	-1.947	124.0395,295.1696,313.1792	$C_{20}H_{25}O_3$-NicA
240[a]	13.14	628.2746	628.2752	$[M+H]^+$	$C_{33}H_{42}O_{11}N$	-1.015	124.0395,311.1640,329.1741,347.1849,434.1971,452.2068,550.2440 ,568.2532,610.2574	$C_{20}H_{25}O_4$-NicA-AcA-B
241	13.14	681.2798	681.2807	$[M+H]^+$	$C_{39}H_{41}O_9N_2$	-1.258	124.0394,295.1694,313.1792,436.2098,558.2481	$C_{20}H_{25}O_3$-2NicA-BzA

242[a]	13.18	438.2264	438.2275	[M+H]+	$C_{26}H_{32}O_5N$	-2.509	124.0394,315.1949	$C_{20}H_{27}O_3$-NicA
243	13.19	574.2432	574.2435	[M+H]+	$C_{33}H_{36}O_8N$	-0.598	124.0394,105.0337,293.1531,311.1633,329.1739,436.2106,452.2188 ,556.2317	$C_{20}H_{25}O_4$-NicA-BzA
244	13.20	552.2584	552.2592	[M+H]+	$C_{31}H_{38}O_8N$	-1.437	124.0394,295.1685,313.1791,418.1975,436.2091	$C_{20}H_{25}O_3$-NicA-B
245	13.25	584.2837	584.2854	[M+H]+	$C_{32}H_{42}O_9N$	-2.924	124.0394,295.1687,313.1792,331.1901,418.2051,436.2113,454.2215 ,496.2323,514.2446,566.2745	$C_{20}H_{25}O_3$-NicA-AcA-A
246[a]	13.27	542.2743	542.2748	[M+H]+	$C_{30}H_{40}O_8N$	-1.002	124.0394,313.1831,494.2291	$C_{20}H_{27}O_4$-NicA-A
247	13.28	634.2640	634.2647	[M+H]+	$C_{35}H_{40}O_{10}N$	-1.061	105.0338,124.0394,293.1528,311.1633,329.1742,434.1943,452.2044 ,494.2131,512.2264,556.2327,574.2410,616.2527	$C_{20}H_{25}O_4$-NicA-BzA-AcA
248	13.30	596.2843	596.2854	[M+H]+	$C_{33}H_{42}O_9N$	-1.859	124.0394,295.1688,313.1793,418.2026,436.2110,454.2196,536.2673 ,554.2712,578.2740	$C_{20}H_{25}O_3$-NicA-C
249[a]	13.30	612.2783	612.2803	[M+H]+	$C_{33}H_{42}O_{10}N$	-3.304	124.0394,295.1689,313.1794,331.1900,436.2111,454.2212,552.2568 ,570.2720	$C_{20}H_{27}O_4$-NicA-B
250[a]	13.34	632.2498	632.2490	[M+H]+	$C_{35}H_{38}O_{10}N$	1.229	124.0394,105.0338,309.1483,327.1587,432.1815,450.1901,554.2148 ,572.2280,614.2379	$C_{20}H_{23}O_4$-NicA-BzA-AcA
251	13.36	584.2848	584.2854	[M+H]+	$C_{32}H_{42}O_9N$	-1.041	124.0394,295.1684,313.1795,331.1894,436.2119,454.2211,496.2331 ,524.2746	$C_{20}H_{25}O_3$-NicA-AcA-A
252	13.37	558.2483	558.2486	[M+H]+	$C_{33}H_{36}O_7N$	-0.589	124.0395,105.0338,295.1680,313.1796,436.2107	$C_{20}H_{25}O_3$-NicA-BzA
253	13.41	576.2568	576.2592	[M+H]+	$C_{33}H_{38}O_8N$	-4.154	124.0394,105.0338,295.1688,313.1797,331.1907,436.2125,454.2209	$C_{20}H_{27}O_4$-NicA-BzA
254	13.43	634.2623	634.2647	[M+H]+	$C_{35}H_{40}O_{10}N$	-0.588	105.0338,124.0394,293.1526,311.1635,329.1743,434.1956,452.2068 ,494.2186,512.2263,556.2325,574.2429,616.2540	$C_{20}H_{25}O_4$-NicA-BzA-AcA
255[a]	13.43	550.2429	550.2435	[M+H]+	$C_{31}H_{36}O_8N$	-1.169	124.0394,293.1519,311.1641,500.2429	$C_{20}H_{23}O_3$-NicA-B
256	13.49	554.2744	554.2748	[M+H]+	$C_{31}H_{40}O_8N$	-0.800	124.0395,297.1847,315.19489,420.2172,438.2269,436.2682	$C_{20}H_{27}O_3$-NicA-B
257	13.51	576.2585	576.2592	[M+H]+	$C_{33}H_{38}O_8N$	-1.204	124.0395,105.0337,295.1688,313.1794,331.1894,436.2113,454.2225	$C_{20}H_{27}O_4$-NicA-BzA
258[a]	13.55	560.2637	560.2643	[M+H]+	$C_{33}H_{38}O_7N$	-1.033	124.0395,105.0338,297.1844,315.1952,420.2145,438.2270,542.2549	$C_{20}H_{27}O_3$-NicA-BzA
259[a]	13.59	612.2781	612.2803	[M+H]+	$C_{33}H_{42}O_{10}N$	-3.630	124.0394,295.1686,313.1795,331.1898,373.2002	$C_{20}H_{25}O_3$-NicA-AcA-B

260	13.59	618.2690	618.2698	$[M+H]^+$	$C_{35}H_{40}O_9N$	-1.226	124.0394,105.0338,295.1685,313.1793,331.1883,436.2098,454.2199 ,496.2327,558.2449	$C_{20}H_{25}O_3$-NicA-BzA-AcA
261	13.61	440.2420	440.2431	$[M+H]^+$	$C_{26}H_{34}O_5N$	-2.661	124.0395,317.2102	$C_{20}H_{29}O_3$-NicA
262	13.63	576.2588	576.2592	$[M+H]^+$	$C_{33}H_{38}O_8N$	-0.683	124.0395,295.1688,313.1801	$C_{20}H_{27}O_4$-NicA-BzA
263[a]	13.64	598.3012	598.3011	$[M+H]^+$	$C_{33}H_{44}O_9N$	0.237	124.0394,297.1844,315.1950,420.2126,438.2253,438.2759, 580.2903	$C_{20}H_{27}O_3$-NicA-C
264[a]	13.65	612.2788	612.2803	$[M+H]^+$	$C_{33}H_{42}O_{10}N$	-2.487	124.0394,295.1688,313.1794,331.1889,418.1996,436.2109,496.2349 ,552.2549	$C_{20}H_{25}O_3$- NicA-AcA-B
265	13.68	513.2471	513.2483	$[M+H]^+$	$C_{29}H_{37}O_8$	-2.327	105.0338,295.1689,313.1793,331.1882,373.2617	$C_{20}H_{27}O_4$-BzA-AcA
266	13.72	574.2432	574.2435	$[M+H]^+$	$C_{33}H_{36}O_8N$	-0.598	124.0394,295.1689,313.1794,436.2114,454.2209	$C_{20}H_{27}O_4$-NicA-2AcA
267[a]	13.72	526.2792	526.2799	$[M+H]^+$	$C_{30}H_{40}O_7N$	-1.385	124.0395,297.1833,315.1951,438.2245,526.2792	$C_{20}H_{27}O_3$-NicA-A
268	13.76	552.2585	552.2592	$[M+H]^+$	$C_{31}H_{38}O_8N$	-1.256	124.0395,295.1682,313.1793	$C_{20}H_{25}O_3$-NicA-B
269	13.78	681.2798	681.2807	$[M+H]^+$	$C_{39}H_{41}O_9N_2$	-1.258	124.0394,105.0338,295.1682,313.1790,436.2113,559.2426	$C_{20}H_{25}O_3$-2NicA-BzA
270	13.78	572.2832	572.2854	$[M+NH_4]^+$	$C_{31}H_{42}O_9N$	-3.859	105.0338,295.1690,313.1794,435.2170	$C_{20}H_{25}O_3$-BzA-2AcA
271	13.80	618.2692	618.2698	$[M+H]^+$	$C_{35}H_{40}O_9N$	-0.903	124.0394,105.0337,295.1681,313.1790,331.1896,436.2102,558.2478	$C_{20}H_{25}O_3$-NicA-BzA-AcA
272[a]	13.82	656.3053	656.3065	$[M+H]^+$	$C_{35}H_{46}O_{11}N$	-1.886	124.0394,295.1688,313.1793,331.1906,418.2022,436.2123,496.2326 ,514.2332,554.2749,578.2737,596.2845,614.2932,638.2908	$C_{20}H_{25}O_3$-NicA-AcA-C
273[a]	13.82	526.2791	526.2799	$[M+H]^+$	$C_{30}H_{40}O_7N$	-1.575	124.0394,297.1842,315.1946,333.2060,456.2358,526.2791	$C_{20}H_{27}O_3$-NicA-A
274	13.92	558.2479	558.2486	$[M+H]^+$	$C_{33}H_{36}O_7N$	-1.306	124.0394,105.0338,295.1689,313.1782,418.1996,436.2094	$C_{20}H_{25}O_3$-NicA-BzA
275	13.92	596.2846	596.2854	$[M+H]^+$	$C_{33}H_{42}O_9N$	-1.355	124.0394,295.1686,313.1793,454.2207,496.2327,536.2610,578.2733	$C_{20}H_{25}O_3$-NicA-C
276[a]	13.93	612.2785	612.2803	$[M+H]^+$	$C_{33}H_{42}O_{10}N$	-2.997	124.0395,295.1692,313.1794,331.1881,436.2111,454.2218,570.2775	$C_{20}H_{25}O_3$-NicA-AcA-B
277	13.93	530.3099	530.3112	$[M+H]^+$	$C_{30}H_{44}O_7N$	-2.506	124.0395,283.2057,301.2161,424.2469	$C_{20}H_{29}O_2$-NicA-X3
278[a]	13.94	526.2788	526.2799	$[M+H]^+$	$C_{30}H_{40}O_7N$	-2.145	124.0395,297.1835,315.1950,456.2339,526.2753	$C_{20}H_{27}O_3$-NicA-A
279[a]	13.94	679.2642	679.2650	$[M+H]^+$	$C_{39}H_{39}O_9N_2$	-1.188	124.0394,105.0338,293.1531,311.1631,434.1956,557.2263	$C_{20}H_{23}O_3$-2NicA-BzA
280[a]	13.96	616.2537	616.2541	$[M+H]^+$	$C_{35}H_{38}O_9N$	-0.662	124.0394,105.0338,293.1538,311.1635,329.1728,434.1970,494.2168	$C_{20}H_{23}O_3$-NicA-BzA-AcA
281	14.02	592.2896	592.2905	$[M+NH_4]^+$	$C_{34}H_{42}O_8N$	-1.509	105.0338,295.1690,313.1793,331.1899,435.2170,453.2267	$C_{20}H_{27}O_4$-2BzA

282	14.03	558.2480	558.2486	$[M+H]^+$	$C_{33}H_{36}O_7N$	-1.127	124.0394,105.0337,295.1688,313.1790,436.2124	$C_{20}H_{25}O_3$-NicA-BzA
283	14.06	618.2681	618.2698	$[M+H]^+$	$C_{35}H_{40}O_9N$	-2.682	124.0395,105.0338,295.1687,313.1793	$C_{20}H_{25}O_3$-NicA-BzA-AcA
284[a]	14.17	722.2985	722.2960	$[M+H]^+$	$C_{42}H_{44}O_{10}N$	3.499	124.0394,105.0338,147.0441,295.1683,313.1794,576.2559	$C_{20}H_{27}O_4$-NicA-BzA-X4
285	14.18	552.2579	552.2592	$[M+H]^+$	$C_{31}H_{38}O_8N$	-2.342	124.0394,295.1691,313.1794 ,331.1906,454.2215	$C_{20}H_{25}O_3$-NicA-B
286[a]	14.19	738.2886	738.2909	$[M+H]^+$	$C_{42}H_{44}O_{11}N$	-3.098	124.0394,105.0337,147.0441,293.1534,311.1683,329.1743,452.2061	$C_{20}H_{27}O_5$-NicA-BzA-X4
287[a]	14.23	612.3152	612.3167	$[M+H]^+$	$C_{34}H_{46}O_9N$	-2.463	124.0395,295.1690,313.1791,524.2629,552.2285	$C_{20}H_{25}O_3$-NicA-2A
288	14.26	554.2735	554.2748	$[M+H]^+$	$C_{31}H_{40}O_8N$	-2.424	124.0395,315.1958	$C_{20}H_{27}O_3$-NicA-B
289	14.30	618.2694	618.2698	$[M+H]^+$	$C_{35}H_{40}O_9N$	-0.579	124.0394,105.0338,295.1683,313.1790,331.1895,436.2138,454.2206,496.2361	$C_{20}H_{25}O_3$-NicA-BzA-AcA
290[a]	14.31	722.2945	722.2960	$[M+H]^+$	$C_{42}H_{44}O_{10}N$	-2.039	124.0394,105.0337,147.0440,295.1688,313.1792,436.2118,576.2583,599.2691	$C_{20}H_{27}O_4$-NicA-BzA-X4
291	14.31	495.2401	495.2377	$[M+H]^+$	$C_{29}H_{35}O_7$	4.786	105.0338,295.1691,313.1791,331.1890	$C_{20}H_{25}O_3$-BzA-AcA
292[a]	14.31	582.3058	582.3061	$[M+H]^+$	$C_{33}H_{44}O_8N$	-1.105	124.0394,281.1897,299.2002,422.2325,494.4600,540.2957	$C_{20}H_{27}O_2$-NicA-C
293[a]	14.33	455.2414	455.2428	$[M+H]^+$	$C_{27}H_{35}O_6$	-3.109	105.0338,297.1841,315.1952,333.2052,437.3414	$C_{20}H_{29}O_4$-BzA
294[a]	14.38	538.2795	538.2799	$[M+H]^+$	$C_{31}H_{40}O_7N$	-0.797	124.0395,299.2000,422.2322	$C_{20}H_{27}O_2$-NicA-B
295	14.41	596.2836	596.2854	$[M+H]^+$	$C_{33}H_{42}O_9N$	-3.033	124.0393,295.1684,313.1793,454.2215,554.2733	$C_{20}H_{25}O_3$-NicA-C
296[a]	14.41	656.3066	656.3065	$[M+H]^+$	$C_{35}H_{46}O_{11}N$	0.095	124.0394,295.1688,313.1807,596.2863	$C_{20}H_{25}O_3$-NicA-AcA-C
297[a]	14.44	560.2638	560.2643	$[M+H]^+$	$C_{33}H_{38}O_7N$	-0.885	124.0395,105.0338,297.1841,315.1950,437.2328	$C_{20}H_{27}O_3$-NicA-BzA
298	14.44	544.2692	544.2694	$[M+H]^+$	$C_{33}H_{38}O_6N$	-0.302	124.0394,105.0338,281.1908,299.2003,422.2360	$C_{20}H_{27}O_2$-NicA-BzA
299	14.48	530.3102	530.3112	$[M+H]^+$	$C_{30}H_{44}O_7N$	-1.941	124.0395,283.2052,301.2159,424.2466,484.2672	$C_{20}H_{29}O_2$-NicA-X3
300[a]	14.49	722.2974	722.2960	$[M+H]^+$	$C_{42}H_{44}O_{10}N$	1.976	124.0394,105.0338,147.0441,295.1687,313.1794,436.2119,558.2486,600.2574	$C_{20}H_{27}O_4$-NicA-BzA-X4
301	14.50	618.2685	618.2698	$[M+H]^+$	$C_{35}H_{40}O_9N$	-2.035	124.0394,105.0338,295.1688,313.1794,331.1895,436.2126,496.2328,558.2507	$C_{20}H_{25}O_3$-NicA-BzA-AcA
302	14.50	558.2482	558.2486	$[M+H]^+$	$C_{33}H_{36}O_7N$	-0.768	124.0395,105.0338,295.1686,313.1793,435.2140	$C_{20}H_{25}O_3$-NicA-BzA
303	14.50	575.2627	575.2639	$[M+H]^+$	$C_{34}H_{39}O_8$	-2.163	105.0338,295.1690,313.1794,435.2138,558.2721	$C_{20}H_{27}O_4$-2BzA

304	14.52	554.2755	554.2748	$[M+H]^+$	$C_{31}H_{40}O_8N$	1.184	124.0394,315.1946,438.2276	$C_{20}H_{27}O_3$-NicA-B
305[a]	14.60	560.2645	560.2643	$[M+H]^+$	$C_{33}H_{38}O_7N$	0.395	124.0395,105.0338,297.1842,315.1952	$C_{20}H_{27}O_3$-NicA-BzA
306[a]	14.61	535.2681	535.2690	$[M+H]^+$	$C_{32}H_{39}O_7$	-1.737	105.0338,295.1685,313.1797	$C_{20}H_{25}O_3$-BzA-X2
307	14.63	552.2576	552.2592	$[M+H]^+$	$C_{31}H_{38}O_8N$	-2.886	124.0394,295.1688,313.1794,436.2094	$C_{20}H_{25}O_3$-NicA-B
308	14.67	554.2727	554.2748	$[M+H]^+$	$C_{31}H_{40}O_8N$	-3.867	124.0395,297.1845,315.1952	$C_{20}H_{27}O_3$-NicA-B
309	14.71	618.2692	618.2698	$[M+H]^+$	$C_{35}H_{40}O_9N$	-0.903	124.0394,105.0338,295.1691,313.1791,331.1893,373.2003,496.2328 ,558.9297	$C_{20}H_{25}O_3$-NicA-BzA-AcA
310[a]	14.74	752.3038	752.3065	$[M+H]^+$	$C_{43}H_{46}O_{11}N$	-3. 639	145.0284,177.0546,124.0394,105.0338,313.1795,436.2116,558.2469	$C_{20}H_{27}O_4$-NicA-BzA-X5
311[a]	14.75	586.3000	586.3011	$[M+NH_4]^+$	$C_{32}H_{44}O_9N$	-1.805	105.0337,295.1690,313.1793,331.1896,435.2128	$C_{20}H_{27}O_4$-BzA-B
312	14.77	558.2480	558.2486	$[M+H]^+$	$C_{33}H_{36}O_7N$	-1.127	124.0394,105.0338,295.1690,313.1794,436.2123	$C_{20}H_{25}O_3$-NicA-BzA
313	14.78	576.2573	576.2592	$[M+H]^+$	$C_{33}H_{38}O_8N$	-3.286	124.0395,105.0338,295.1689,313.1794,331.1879,436.2117,454.2222	$C_{20}H_{27}O_4$-NicA-BzA
314[a]	14.79	646.3004	646.3011	$[M+H]^+$	$C_{37}H_{44}O_9N$	-1.019	124.0395,105.0338,295.1685,313.1793,436.2127,558.2479	$C_{20}H_{25}O_3$-NicA-BzA-A
315	14.83	544.2684	544.2694	$[M+H]^+$	$C_{33}H_{38}O_6N$	-1.772	124.0394,105.0338,281.1895,299.2000,422.2329	$C_{20}H_{27}O_2$-NicA-BzA
316[a]	14.89	560.2639	560.2643	$[M+H]^+$	$C_{33}H_{38}O_7N$	-0.676	124.0394,105.0338,297.1840,315.1949,438.2271	$C_{20}H_{27}O_3$-NicA-BzA
317	14.91	572.2832	572.2854	$[M+NH_4]^+$	$C_{31}H_{42}O_9N$	-3.859	105.0338,295.1690,313.1794,331.1899,373.2006,435.2166	$C_{20}H_{25}O_3$-BzA-2AcA
318	14.96	575.2626	575.2639	$[M+H]^+$	$C_{34}H_{39}O_8$	-2.337	105.0338,295.1689,313.1794,435.2160,453.2267	$C_{20}H_{27}O_4$-2BzA
319[a]	14.97	652.3109	652.3116	$[M+H]^+$	$C_{36}H_{46}O_{10}N$	-1.108	124.0393,295.1687,313.1787,472.3964,490.4098,536.2615,634.2975	$C_{20}H_{25}O_3$-NicA-B-X2
320[a]	15.00	435.2193	435.2166	$[M+H]^+$	$C_{27}H_{31}O_5$	6.230	105.0338,295.1673,313.1794	$C_{20}H_{25}O_3$-BzA
321[a]	15.05	646.3002	646.3011	$[M+H]^+$	$C_{37}H_{44}O_9N$	-1.638	124.0394,105.0337,295.1676,313.1797,524.2620,576.2568	$C_{20}H_{25}O_3$-NicA-BzA-A
322[a]	15.13	560.2639	560.2643	$[M+H]^+$	$C_{33}H_{38}O_7N$	-0.676	124.0394,105.0338,297.1837,315.1949,438.2266	$C_{20}H_{27}O_3$-NicA-BzA
323	15.18	558.2482	558.2486	$[M+H]^+$	$C_{33}H_{36}O_7N$	-0.768	124.0394,105.0338,295.1694,313.1794,436.2114	$C_{20}H_{25}O_3$-NicA-BzA
324[a]	15.19	712.3104	712.3116	$[M+H]^+$	$C_{41}H_{46}O_{10}N$	-1.717	124.0394,105.0338,309.1856,327.1949,345.2056,450.2263,468.2374 ,590.2743	$C_{21}H_{29}O_4$-NicA-2BzA
325[a]	15.21	658.3010	658.3011	$[M+H]^+$	$C_{38}H_{44}O_9N$	-0.089	124.0394,105.0338,297.1843,315.1951,420.2169,612.2578,640.2881	$C_{20}H_{25}O_2$-NicA-BzA-B
326	15.22	551.2628	551.2639	$[M+H]^+$	$C_{32}H_{39}O_8$	0.619	105.0338,295.1692,313.1785,435.1790,495.2383	$C_{20}H_{25}O_3$- BzA-B
327[a]	15.31	652.3102	652.3116	$[M+H]^+$	$C_{36}H_{46}O_{10}N$	-2.181	124.0393,295.1685,313.1790,536.2637,634.3003	$C_{20}H_{25}O_3$-NicA-B-X2

328	15.31	433.1997	433.2010	$[M+H]^+$	$C_{27}H_{29}O_5$	-2.887	105.0338,311.1632	$C_{20}H_{23}O_3$-BzA
329	15.31	493.2206	493.2221	$[M+H]^+$	$C_{29}H_{33}O_7$	-3.000	105.0338,311.1634,329.1736,433.2007	$C_{20}H_{23}O_3$-BzA-AcA
330[a]	15.31	646.3004	646.3011	$[M+H]^+$	$C_{37}H_{44}O_9N$	-1.019	124.0394,105.0338,295.1691,313.1793,436.2107,558.2478	$C_{20}H_{25}O_3$-NicA-BzA-A
331	15.32	633.2677	633.2694	$[M+H]^+$	$C_{36}H_{41}O_{10}$	-2.722	105.0338,311.1635,329.1736,433.2010,451.1745,615.2935	$C_{20}H_{25}O_4$-2BzA-AcA
332	15.35	568.2889	568.2878	$[M+NH_4]^+$	$C_{32}H_{42}O_8N$	-2.804	105.0337,295.1688,313.1794,417.2251,435.2162	$C_{20}H_{25}O_3$-BzA-B
333[a]	15.37	511.2313	511.2326	$[M+H]^+$	$C_{29}H_{35}O_8$	-2.239	105.0338,311.1630,329.1738,	$C_{20}H_{25}O_4$-BzA-AcA
334[a]	15.41	439.2467	439.2479	$[M+H]^+$	$C_{27}H_{35}O_5$	-2.733	105.0338,299.2003,421.3026,	$C_{20}H_{29}O_3$-BzA
335	15.42	592.2888	592.2905	$[M+NH_4]^+$	$C_{34}H_{42}O_8N$	-2.860	105.0338,295.1683,313.1794,331.1898,435.2168,575.2657	$C_{20}H_{27}O_4$-2BzA
336	15.43	634.2983	634.3011	$[M+NH_4]^+$	$C_{36}H_{44}O_9N$	-4.349	105.0338,295.1686,313.1792,331.1902,435.2154,	$C_{20}H_{25}O_3$-2BzA-AcA
337[a]	15.45	644.3044	644.3065	$[M+NH_4]^+$	$C_{34}H_{46}O_{11}N$	-3.318	105.0338,293.1530,311.1636,329.1742,433.1998,451.2103,511.2316 ,609.2707	$C_{20}H_{25}O_4$-BzA-AcA-B
338	15.45	609.2721	609.2694	$[M+H]^+$	$C_{34}H_{41}O_{10}$	4.392	105.0338,311.1643,329.1740,433.2021,451.2139,451.2139,549.2499 ,591.402	$C_{20}H_{23}O_3$-BzA-AcA-B
339	15.47	575.2628	575.2639	$[M+H]^+$	$C_{34}H_{39}O_8$	-1.990	105.0338,295.1688,313.1792,331.1896,435.2143	$C_{20}H_{27}O_4$-2BzA
340[a]	15.48	560.2646	560.2643	$[M+H]^+$	$C_{33}H_{38}O_7N$	0.573	124.0395,105.0338,297.1851,315.1945	$C_{20}H_{27}O_3$-NicA-BzA
341	15.55	680.2845	680.2854	$[M+H]^+$	$C_{40}H_{42}O_9N$	-1.335	124.0394,105.0338,295.1683,313.1792,436.2120,558.2499	$C_{20}H_{25}O_3$- NicA-2BzA
342[a]	15.55	654.3267	654.3274	$[M+H]^+$	$C_{36}H_{48}O_{10}N$	-0.876	124.0394,297.1845,315.1953,482.3555,520.2679,538.2819,572.6536 ,636.3153	$C_{20}H_{27}O_3$-NicA-B-X2
343[a]	15.56	660.3174	660.3167	$[M+H]^+$	$C_{38}H_{46}O_9N$	1.047	105.0338,124.0394,297.1846,315.1948,420.2180,438.2234,514.2217 ,642.3066	$C_{20}H_{27}O_3$-NicA-BzA-X2
344	15.63	592.2894	592.2905	$[M+NH_4]^+$	$C_{34}H_{42}O_8N$	-1.846	105.0338,295.1688,313.1793,331.1901	$C_{20}H_{27}O_4$-2BzA
345	15.65	680.2842	680.2854	$[M+H]^+$	$C_{40}H_{42}O_9N$	-1.776	124.0394,105.0338,295.1684,313.1792,436.2097,558.2484	$C_{20}H_{25}O_3$-NicA-2BzA
346	15.66	451.2132	451.2115	$[M+H]^+$	$C_{27}H_{31}O_6$	3.734	105.0338,293.1592,311.1640,329.1742	$C_{20}H_{25}O_4$-BzA
347	15.74	551.2642	551.2639	$[M+H]^+$	$C_{32}H_{39}O_8$	0.463	105.0338,295.1685,313.1795	$C_{20}H_{25}O_3$-BzA-B
348[a]	15.74	658.2999	658.3011	$[M+H]^+$	$C_{38}H_{44}O_9N$	-1.760	124.0394,105.0338,295.1686,313.1793,417.2050,518.2521,536.2629 ,640.297	$C_{20}H_{25}O_3$-NicA-BzA-X2

349	15.76	451.2116	451.2115	$[M+H]^+$	$C_{27}H_{31}O_6$	0.188	105.0339,293.1533,311.1639,329.1745	$C_{20}H_{25}O_4$-BzA
350	15.76	557. 2521	557.2534	$[M+H]^+$	$C_{34}H_{37}O_7$	-2.297	105.0339,295.1689,313.1796,435.2123,475.4920	$C_{20}H_{25}O_3$-2BzA
351	15.78	608. 2842	6082854	$[M+NH_4]^+$	$C_{34}H_{42}O_9N$	-1.986	105.0338,293.1531,311.1637,329.1742,347.1850,451.2112	$C_{20}H_{27}O_5$-2BzA
352[a]	15.80	678.2697	678.2698	$[M+H]^+$	$C_{40}H_{40}O_9N$	-0.086	124.0396,105.0339,293.1535,311.1637,555.2380	$C_{20}H_{23}O_3$-NicA-2BzA
353[a]	15.88	435.2148	435.2166	$[M+H]^+$	$C_{27}H_{31}O_5$	-4.137	105.0338,295.1690,313.1795	$C_{20}H_{25}O_3$-BzA
354	15.91	557.2508	557.2534	$[M+H]^+$	$C_{34}H_{37}O_7$	-4.630	105.0338,295.1689,313.1794	$C_{20}H_{25}O_3$-2BzA
355	15.96	680.2844	680.2854	$[M+H]^+$	$C_{40}H_{42}O_9N$	-1.482	124.0394,105.0338,295.1689,313.1791,436.2112,558.2475	$C_{20}H_{25}O_3$-NicA-2BzA
356	16.00	568.2891	568.2878	$[M+NH_4]^+$	$C_{32}H_{42}O_8N$	-2.452	105.0338,295.1688,313.1793,435.2164,453.1892,551.2634	$C_{20}H_{25}O_3$-BzA-B
357[a]	16.03	660.3156	660.3167	$[M+H]^+$	$C_{38}H_{46}O_9N$	-1.679	105.0338,124.0394,297.1847,315.1951,538.2787,642.3064	$C_{20}H_{27}O_3$-NicA-BzA-X2
358[a]	16.05	722.2936	722.2960	$[M+H]^+$	$C_{42}H_{44}O_{10}N$	-3.285	124.0394,105.0546,131.0492,293.2533,311.1631,329.1743,452.2061 ,624.4337	$C_{20}H_{27}O_5$-NicA-BzA-X6
359	16.05	573.2454	573.2483	$[M+H]^+$	$C_{34}H_{37}O_8$	-4.562	105.0338,293.1514,311.1636,329.1742,433.1996,457.1981,498.2237	$C_{20}H_{25}O_4$-2BzA
360	16.06	680.2845	680.2854	$[M+H]^+$	$C_{40}H_{42}O_9N$	-1.335	124.0394,105.0338,295.1683,313.1793,436.2098,558.2469	$C_{20}H_{25}O_3$-NicA-2BzA
361	16.08	617.2733	617.2745	$[M+H]^+$	$C_{36}H_{41}O_9$	-1.959	105.0338,295.1688,313.1793,331.1898,373.1999,417.2058,435.2155 ,477.2267	$C_{20}H_{25}O_3$-2BzA-AcA
362	16.17	595. 2886	595.2902	$[M+H]^+$	$C_{34}H_{43}O_9$	-2.619	105.0338,115.0755,295.1688,313.1793,417.2057,435.2165	$C_{20}H_{25}O_3$-BzA-C
363	16.19	592. 2892	592.2905	$[M+NH_4]^+$	$C_{34}H_{42}O_8N$	-2.184	105.0338,295.1687,313.1790,331.1898,435.2158,453.2220,575.2679	$C_{20}H_{27}O_4$-2BzA
364[a]	16.26	721.2980	721.3007	$[M+H]^+$	$C_{43}H_{45}O_{10}$	-3.776	105.0338,147.0440,295.1681,313.1794, 435.2176,623.4336,645.2123	$C_{20}H_{27}O_4$-2BzA-X4
365[a]	16.28	570. 3050	570.3061	$[M+NH_4]^+$	$C_{32}H_{44}O_8N$	-2.005	105.0338,297.1845,315.1953,419.2195,437.2311	$C_{20}H_{27}O_3$-BzA-B
366	16.29	617.2728	617.2745	$[M+H]^+$	$C_{36}H_{41}O_9$	-2.769	105.0338,295.1687,313.1792,331.1900,355.1894	$C_{20}H_{25}O_3$-2BzA-AcA
367	16.31	680.2848	680.2854	$[M+H]^+$	$C_{40}H_{42}O_9N$	-0.894	124.0395,105.0338,295.1683,313.1795,436.2151,662.4343	$C_{20}H_{25}O_3$-NicA-2BzA
368	16.40	592. 2891	592.2905	$[M+NH_4]^+$	$C_{34}H_{42}O_8N$	-2.353	105.0338,295.1688,313.1793,331.1896,435.2160	$C_{20}H_{27}O_4$-2BzA
369	16.68	574.2785	574.2799	$[M+NH_4]^+$	$C_{34}H_{40}O_7N$	-2.488	105.0338,295.1689,313.1795,435.2096	$C_{20}H_{25}O_3$-2BzA
370[a]	16.71	672.3358	672.3378	$[M+NH_4]^+$	$C_{36}H_{50}O_{11}N$	-3.031	105.0338,115.0755,295.1689,313.1793,331.1880,435.2170,477.2274 ,612.1678,654.4383	$C_{20}H_{25}O_3$-BzA-AcA-C

371	16.77	576.2943	576.2956	$[M+NH_4]^+$	$C_{34}H_{42}O_7N$	-2.219	105.0338,297.1844,315.1950,437.2316,559.2676	$C_{20}H_{27}O_3$-2BzA
372[a]	16.83	560.2994	560.3007	$[M+NH_4]^+$	$C_{34}H_{42}O_6N$	-2.257	105.0338,281.1900,299.2002,403.2266,421.2355,543.2731	$C_{20}H_{27}O_2$-2BzA
373	16.83	680.2853	680.2854	$[M+H]^+$	$C_{40}H_{42}O_9N$	-0.159	124.0394,105.0339,295.1689,313.1794,435.2165,558.2464	$C_{20}H_{25}O_3$-NicA-2BzA
374	16.94	617. 2724	617.2745	$[M+H]^+$	$C_{36}H_{41}O_9$	-3.417	105.0338,295.1689,313.1791,331.1894,435.2161	$C_{20}H_{25}O_3$-2BzA-AcA
375	16.95	576.2936	576.2956	$[M+NH_4]^+$	$C_{34}H_{42}O_7N$	-3.434	105.0338,297.1846,315.1951, 437.2330	$C_{20}H_{27}O_3$-2BzA
376	16.99	680.2845	680.2854	$[M+H]^+$	$C_{40}H_{42}O_9N$	-1.335	105.0338,124.0394,295.1691,313.1793,435.2159,463.2425,558.2473 ,662.2675	$C_{20}H_{25}O_3$- NicA-2BzA
377	17.21	576.2952	576.2956	$[M+NH_4]^+$	$C_{34}H_{42}O_7N$	-0.658	105.0338,297.1845,315.1951,437.2332	$C_{20}H_{27}O_3$-2BzA
378	17.32	576.2952	576.2956	$[M+NH_4]^+$	$C_{34}H_{42}O_7N$	-0.658	105.0338,297.1843,315.1951,437.2328	$C_{20}H_{27}O_3$-2BzA
379[a]	17.69	696.3195	696.3167	$[M+NH_4]^+$	$C_{41}H_{46}O_9N$	4.009	105.0339,295.1692,313.1795,331.1894,417.2027,435.2162,557.2539 ,679.2894	$C_{20}H_{25}O_3$-3BzA
380[a]	17.80	696.3155	696.3167	$[M+NH_4]^+$	$C_{41}H_{46}O_9N$	-1.735	105.0338,295.1688,313.1793,331.1903,435.2161,557.2585	$C_{20}H_{25}O_3$-3BzA
381	18.16	576.2943	576.2956	$[M+NH_4]^+$	$C_{34}H_{42}O_7N$	-2.219	105.0337,297.1841,315.1949,437.2337	$C_{20}H_{27}O_3$-2BzA

Glc: $C_6H_{12}O_6$; A: $C_4H_8O_2$; B: $C_5H_8O_3$; C: $C_7H_{12}O_4$; X1: $C_3H_8O_3$; X2: $C_5H_8O_2$; X3: $C_4H_{10}O_3$; X4: $C_9H_6O_2$; X5: $C_{10}H_8O_3$; X6: C_9H_6O.

a: compounds with new molecular mass never reported in *Scutellaria barbata*.
